# Flu Shots Unveiled: A Global Systematic Review of Healthcare Providers’ Uptake of, Perceptions, and Attitudes toward Influenza Vaccination

**DOI:** 10.3390/vaccines11121760

**Published:** 2023-11-27

**Authors:** Latefa Ali Dardas, Obada Al-leimon, Abdel Rahman Jaber, Mohammed Saadeh, Ahmad Al-leimon, Ahmad Al-Hurani, Abdul-Raheem Jaber, Omer Aziziye, Fadi Al-salieby, Mohammad Aljahalin, Brittney Van de Water

**Affiliations:** 1 School of Nursing, The University of Jordan, Amman 11942, Jordan; 2 School of Medicine, The University of Jordan, Amman 11942, Jordan; 3 William F. Connell School of Nursing, Boston College, Boston, MA 02467, USA

**Keywords:** attitudes, flu, healthcare providers, immunization, influenza, public health, vaccine uptake, vaccine hesitancy

## Abstract

Background and Purpose: Influenza, with its potential for widespread transmission and significant health repercussions for individuals and populations, demands the immediate implementation of effective preventive measures. Vaccination stands as a long-standing evidence-based strategic approach to bolster immunity, especially for healthcare providers at heightened risk due to repeated exposure. Nevertheless, studies indicate a variance in adherence to recommended vaccination protocols and a notable prevalence of hesitancy and negative attitudes toward influenza vaccination among this critical group globally. Recognizing the multifaceted nature of attitudes is essential for the development of targeted interventions and strategies tailored to address the specific concerns and motivations of healthcare providers. To this end, this study synthesized the evidence gathered from an exhaustive systematic review of studies on healthcare providers’ uptake of and perceptions and attitudes toward influenza vaccination. Methods: A systematic literature search was conducted across the databases PubMed, CINAHL, PsycINFO, Scopus, Web of Science, and EMBASE. The review adhered to PRISMA guidelines, using Covidence for screening. The process involved 4970 references, with 2684 screened after duplicate removals and 1891 excluded, leaving 793 full texts evaluated, resulting in a final 368 selected references for analysis. Due to the considerable heterogeneity observed among the studies, a narrative synthesis method was employed. Results: Five themes emerged from the systematic review’s analysis, offering a multifaceted perspective on healthcare providers’ attitudes toward influenza vaccination: (1) fostering positive views: factors promoting attitudes toward influenza vaccines; (2) navigating hesitancy: barriers and challenges to attitudes on influenza vaccines; (3) empowering change: interventions and their impact on healthcare providers’ attitudes; (4) pandemic overlap: intersecting attitudes toward influenza and COVID-19 vaccines; and (5) twin challenges: the impact of mandatory policy on attitudes and influenza vaccination. Conclusions: Healthcare providers’ attitudes toward influenza vaccination are complex and influenced by intrinsic and extrinsic motivations, barriers, demographics, organizational factors, interventions, pandemic contexts, and policy considerations. Effective strategies for promoting influenza vaccination should be multifaceted, adaptable, and tailored to address these interconnected aspects, ultimately contributing to improved vaccination rates and public health outcomes.

## 1. Introduction

Despite advancements in healthcare, communicable diseases persist as a global health threat. Recent outbreaks like Ebola, SARS, and COVID-19 have highlighted the need for global cooperation and coordination in controlling and preventing the spread of disease. Influenza is a major communicable disease that can spread easily, causing significant morbidity and mortality annually. It is estimated that influenza epidemics result in up to 5 million severe cases of disease and up to 650,000 respiratory deaths around the globe each year [1]. In the United States alone, the Centers for Disease Control and Prevention (CDC, 2022) estimated that influenza has resulted in from 9 to 41 million illnesses, from 140,000 to 710,000 hospitalizations, and from 12,000 to 52,000 deaths annually between 2010 and 2020 [2].

Vaccination against influenza stands as the best preventive approach available due to its remarkable effectiveness in reducing the spread and impact of the disease. Unlike other preventive measures, vaccines offer long-lasting protection with efficacy extending across multiple influenza strains, adapting to the virus’s variability [3,4,5,6,7]. Additionally, this preventive approach contributes to economic stability by reducing both direct and indirect financial burdens and upholds the healthcare system’s capacity to effectively manage patient needs [8,9,10,11,12].

Healthcare providers (HCPs) face an elevated risk of contracting the influenza virus due to their heightened ongoing exposure to patients. This increased exposure places them in a vulnerable position not only for their own health but also for potentially transmitting the virus to their patients, colleagues, and vulnerable individuals within the community [13,14]. Therefore, vaccines play an indispensable role in safeguarding the health and well-being of HCPs. By receiving vaccinations, HCPs not only fortify their own immune system but also contribute to the creation of a protective barrier within healthcare settings [13]. Furthermore, HCPs serve as role models within their communities, setting an example for patients and the general public regarding the importance of vaccination. Their proactive engagement with vaccine uptake not only ensures their own health but also establishes a culture of prevention and responsibility that extends to the broader population [15,16]. However, despite compelling reasons for HCPs to receive influenza vaccination [17,18], studies have revealed that not all HCPs adhere to recommended vaccination protocols, and a significant portion exhibit resistance and negative attitudes toward vaccination [19,20].

The phenomenon of vaccine hesitancy traces its origins back to the early stages of vaccination introduction and has garnered increased attention, particularly in light of the ongoing COVID-19 pandemic [21]. This concept encompasses a reluctance or hesitation to accept vaccines, despite readily accessible vaccination services [22]. The significance of this issue extends beyond individual choices, as vaccine hesitancy has the potential to undermine public health efforts, particularly in achieving the levels of immunity necessary for disease control and eradication at the population level. Attempts to delve into the complexities of vaccine hesitancy have underscored the significance of various psychological, sociocultural, political, and media-related factors [20,23,24]. In light of the intricate interplay of these factors, a comprehensive exploration of vaccine hesitancy becomes paramount. This is particularly crucial when considering distinct groups, such as HCPs, who possess their own set of perspectives, motivations, and concerns that can significantly differ from those of the general public.

While several existing reviews have explored aspects related to influenza vaccination among healthcare workers, they often exhibit limitations in their scope and depth, leaving crucial gaps in the literature. These reviews primarily focused on specific aspects, such as interventions to improve vaccine uptake [25], challenges and solutions in increasing vaccination coverage [26], campaign strategies within restricted geographical contexts [27], and insights only until 2015, excluding post-pandemic developments [28]. Additionally, some reviews centered on narrow topics like healthcare workers’ attitudes toward mandatory vaccination [29] or the pandemic’s effect on vaccination intention [30].

Recognizing the gaps in current research, the purpose of this study is to synthesize the evidence gleaned from an exhaustive systematic review of studies focused on HCPs’ attitudes toward influenza vaccination. This systematic review takes a comprehensive approach, synthesizing findings from studies across various research designs and offering a broader geographical perspective and up-to-date insights. By understanding the unique attitudes and perceptions of HCPs, the study seeks to contribute to strategies that enhance influenza vaccine uptake within this specific group. These efforts ultimately hold the potential to improve patient care and bolster public health initiatives.

## 2. Materials and Methods

### 2.1. Literature Search

We initiated our search by examining the Database of Abstracts of Reviews of Effects (DARE) and the Cochrane Database of Systematic Reviews (CDSR) to identify any analogous reviews in existence. Subsequently, we designated the databases PubMed, CINAHL, PsycINFO, Scopus, Web of Science, and EMBASE as our primary sources of data for this comprehensive review. In our pursuit of scholarly materials, we adopted an all-encompassing methodology by employing an assortment of databases and employing diverse variations of search terms relating to HCPs’ attitudes toward influenza vaccines. Collaborating with an academic health center reference librarian, we formulated a combination of index and MeSH terms tailored to align with the requisites of each individual database (Supplementary document S1).

To meet the criteria for eligibility, each study was required to (1) encompass HCPs as the targeted population, encompassing doctors, nurses, pharmacists, and other relevant practitioners, and (2) focus primarily on HCPs’ attitudes toward the influenza vaccine. This encompassed a thorough examination of elements such as intent to vaccinate, vaccine hesitancy, prevailing challenges, factors facilitating vaccine acceptance, relationships between knowledge, attitudes, and practices, as well as interventions and policies aimed at fostering positive attitudes. Inclusivity was a guiding principle, as no constraints were imposed concerning the type of study, publication date, type of publication, or geographical region. Studies that did not involve healthcare providers, had ineligible outcomes, were not in English, or were not original research (e.g., review/meta-analysis articles, editorial letters, commentaries, perspective articles, theses, or conference reports) were excluded from the systematic review.

We meticulously adhered to the tenets outlined in the Preferred Reporting Items for the Systematic Reviews and Meta-Analyses (PRISMA) statement [31] throughout the entirety of the identification, selection, and assessment processes for the studies included in this review. This screening procedure was executed using the systematic review platform Covidence [32]. Each study deemed eligible was subjected to a double review against the established inclusion criteria by two independent reviewers. For systematic abstraction and documentation, we devised a structured matrix, thereby constructing a comprehensive database replete with intricate details pertaining to each record.

### 2.2. Study Extraction

**Figure 1:**
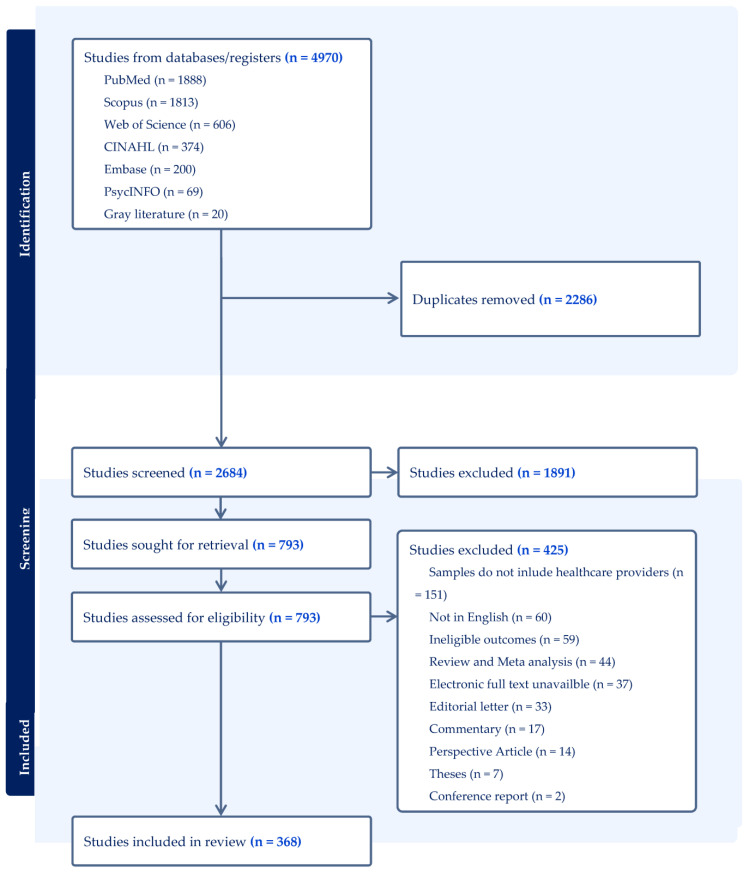
The PRISMA diagram.

In its entirety, the review process involved the handling of 4970 references extracted from various databases. From this initial pool, 2286 duplicate entries were removed, yielding a set of 2684 studies that underwent preliminary screening based on title and abstract. Subsequently, through this screening process, 1891 studies were excluded, leaving a subset of 793 studies that were further evaluated for full-text eligibility. This latter stage of assessment led to the exclusion of an additional 424 studies, resulting in a final selection of 368 studies for comprehensive analysis. Figure 1 presents the PRISMA diagram detailing the stages of inclusion and exclusion. To ensure stringent adherence to the study inclusion criteria, all 368 chosen records underwent a re-evaluation by two independent researchers, and a third reviewer was consulted when consensus could not be reached. This approach involved a comprehensive discussion and examination of the differing assessments with the two independent reviewers. This discussion aimed to identify the specific points of disagreement and foster a consensus through dialogue, enhancing the reliability and validity of the eligibility assessments. The included studies, each possessing its distinct descriptive characteristics, are comprehensively detailed in Supplementary document S2.

### 2.3. Quality Assessment

**Figure 2:**
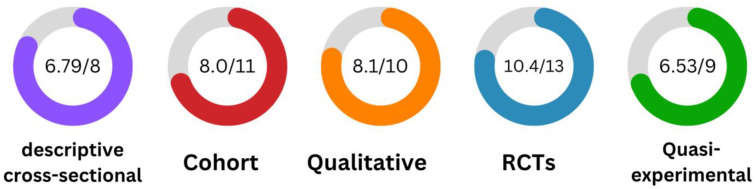
Quality assessment average scores.

We conducted a comprehensive quality assessment of the included studies (n = 368) to ensure the rigor and trustworthiness of the evidence included in our systematic review. To achieve this, we utilized a range of critical appraisal tools developed by the Joanna Briggs Institute (JBI), which were selected based on the specific study types included in our review. Figure 2 presents an overview of the quality assessment scores for all included articles based on the utilized methods. The quality assessment results for each study design, while not directly comparable due to differences in assessment tools and scoring criteria, offer valuable insights. Descriptive cross-sectional studies demonstrated reasonably high methodological quality, cohort studies exhibited commendable rigor, qualitative studies displayed robustness, RCTs maintained the highest level of quality, and quasi-experimental studies showed a relatively good level of adherence to their criteria. A detailed examination of the quality assessment results for each individual study is available in supplementary document S2.

### 2.4. Narrative Synthesis

Due to the considerable heterogeneity observed among the studies incorporated into our comprehensive evidence synthesis, encompassing variations in research scope and methodologies, the application of a traditional meta-analysis approach was rendered unfeasible. Consequently, we opted for an alternative approach, employing a narrative synthesis method [33] to amalgamate and interpret the findings obtained from the extensive 368-corpus dataset. This qualitative synthesis method allowed us to systematically examine and describe the individual study findings, facilitating a comprehensive understanding of the collective evidence within the 368 studies. Through narrative synthesis, we were able to construct a cohesive and informative narrative that sheds light on the complex interplay of factors and the overall implications of the diverse research findings, ultimately offering valuable insights for the research objectives and informing future investigations and interventions in this area.

## 3. Results

### 3.1. Study Characteristics

**Figure 3:**
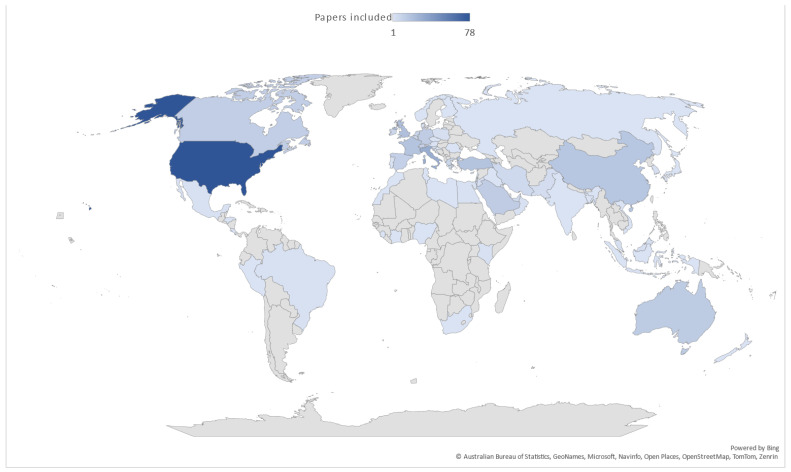
Regional distribution of articles published on healthcare providers’ attitudes toward influenza vaccination.

The synthesis incorporated a comprehensive set of 368 studies that met the stringent inclusion criteria, spanning over thirty years of articles from 1989 to 2023. Notably, the geographic distribution of these studies reflects a broad spectrum of global perspectives, with a substantial portion originating from Northern, Western, and Southern European nations, constituting 163 out of the total. A significant contribution also came from the United States, with 78 publications. Additionally, the Middle East and North Africa yielded 49 publications, while Eastern Asia contributed 30. In contrast, there was relatively limited representation from the African region, consisting of just six publications, and Eastern Europe provided five. Figure 3 visually illustrates the geographic dispersion of the 368 papers. Regarding study designs, the majority, encompassing 308 out of 368, employed descriptive methodologies. Alongside these, 35 papers followed experimental designs. In terms of research methods, the predominant approach was quantitative, with the use of standardized questionnaires evident in the majority of studies (343 out of 368). A smaller subset, consisting of 18 publications, employed qualitative designs, and seven embraced a mixed-methods approach, emphasizing the diversity of research techniques employed. There was also substantial variation in sample sizes across the studies, spanning a wide range from 10 to 368,696 participants, with a median participant count of 547 and an interquartile range of 1004.25 (range 231.5–1235.75). This breadth of data sources and methodologies enhances the robustness and comprehensiveness of our synthesis.

### 3.2. Exploring Key Themes: HCPs’ Attitudes toward Influenza Vaccination

This systematic review unveiled a comprehensive landscape of insights revolving around HCPs’ attitudes toward influenza vaccination. The analysis of a diverse array of sources led to the emergence of five distinct themes that collectively contribute to a holistic understanding of this crucial subject (Table 1). Because there was a substantial quantity of articles involved in the analysis, we incorporated the citation count for each finding within the text. We have also furnished a supplementary document that cites the articles specifically relevant to the findings (Supplementary document S3).

#### 3.2.1. Fostering Positive Views: Factors Promoting Attitudes toward Influenza Vaccines

The review illuminated multifaceted factors that act as catalysts in fostering positive attitudes among HCPs toward influenza vaccines. These encompassed an assortment of intrinsic and extrinsic motivators that collectively highlight the motivations, perceptions, and concerns affecting HCPs’ decisions to receive flu vaccination. It was noted that HCPs’ motivations for receiving influenza vaccinations are consistently centered around self-protection (with reported rates ranging from 53.4% to 87%), patient protection (with reported rates ranging from 31% to 63%), and family protection. These reasons are frequently cited across various studies (n = 272).

The review also revealed that HCPs are motivated by concerns about transmitting influenza specifically to vulnerable patients (with reported rates ranging from 21% to 36%), their families, and themselves. Additionally, HCPs’ beliefs in the vaccine’s effectiveness and its role in preventing the spread of influenza, minimizing viral reservoirs, and reducing hospital visits significantly influence their decision to be vaccinated (n = 270).

Furthermore, factors such as being older, spending a longer time in the profession, being vaccinated before, possessing correct knowledge about vaccine efficacy and safety (encompassing beliefs in proven vaccine efficacy and dispelling misconceptions, such as links between vaccines and autism), as well as professional responsibilities and the perception of vaccination as being a fundamental part of their obligation to ensure patient safety and maintain optimal healthcare delivery, together promoted HCPs’ vaccination decisions.

At the level of healthcare settings, the presence of peer support, organizational culture, and institutional policies appeared to be significant in promoting vaccination among HCPs (n = 265). Further, the availability of free vaccines and workplace access facilitated higher vaccination rates (n = 11). Workplace vaccination campaigns and recommendations by leaders (n = 3) and specific training about vaccination campaigns (n = 2) also promoted uptake.

In summary, HCPs’ motivations for influenza vaccination encompass self-protection, patient protection, and family protection. The belief in vaccine effectiveness, along with concerns about transmitting influenza, also significantly influence their decision. Factors like age, vaccination history, knowledge, and professional responsibilities contribute to their vaccination choices. Peer support, organizational culture, and institutional policies have a pivotal role in promoting vaccination within this group.

#### 3.2.2. Navigating Hesitancy: Barriers and Challenges to Attitudes on Influenza Vaccines

Counterbalancing the positive aspects, this theme delved into the factors that serve as barriers to fostering favorable attitudes. Issues such as fear of side effects (rates ranged from 13% to 63%) (n = 175), concerns related to vaccine efficacy (rates ranged from 9% to 56%) (n = 152), lack of time to be vaccinated (rates ranged from 22% to 47%) (n = 64), beliefs that they do not need to obtain the vaccine as they are healthy (rates ranged from 9% to 35%) (n = 29), perception of not being at risk/being at low risk of contracting the infection (ranging from 2% to 32%) (n = 52), beliefs that vaccination is not necessary (ranging from 23% to 53%) (n = 24), beliefs that influenza is not a serious illness (ranging from 25% to 58%) (n = 39), and beliefs that the vaccine causes illness (ranging from 16% to 38.5%) (n = 39) were most commonly repeated in the literature as major contributors to vaccine hesitancy.

The review also revealed that HCPs are hindered from taking the vaccine because of disliking injections/fear of pain (ranging from 11% to 35%) (n = 62), and fear of local and systemic allergic reactions related to the vaccine (n = 21). System-related barriers including expensive costs (n = 27) and the lack of availability of the vaccine (n = 38) were consistently reported throughout the literature as well. Furthermore, demographic factors such as being younger and female were related to less uptake of the influenza vaccine (n = 8). Also, breastfeeding and having a current pregnancy were reported as barriers to obtaining the vaccine (n = 10) as well as having a chronic illness, having a medical contraindication, or lacking a medical indication for vaccination (n = 24). Some HCPs did not take the vaccine because of being against vaccination (i.e., anti-vaccination) in general (n = 14).

In summary, several factors were identified as major impediments to fostering positive attitudes. These included concerns about vaccine side effects, vaccine efficacy, lack of time for vaccination, the belief that one is healthy and does not need the vaccine, the perception of being at low risk for infection, the belief that vaccination is unnecessary, and the fear that the vaccine itself could cause illness. HCPs also faced barriers, such as a dislike of injections or fear of pain, as well as concerns about allergic reactions related to the vaccine. System-related issues, like the cost and availability of the vaccine, were commonly cited obstacles. Demographic factors, such as younger age and female gender, were associated with lower vaccine uptake. Overall, these findings highlight a range of factors contributing to vaccine hesitancy in the context of influenza vaccination.

#### 3.2.3. Empowering Change: Interventions and Their Impact on HCPs’ Attitudes

The systematic analysis revealed a spectrum of interventions designed to cultivate positive attitudes (n = 38). These interventions were categorized into three primary categories, each offering a unique approach employed to address the challenges of promoting influenza vaccination, cultivating positive attitudes, and increasing awareness of the value of influenza vaccination among HCPs. The categories included campaigns, educational interventions, and mobile art programs.

Tested vaccination campaigns (n = 30) were characterized by the application of multiple strategies aimed at bolstering vaccine coverage among the HCP population. These strategies include the provision of free vaccines (n = 13), effectively reducing financial barriers to vaccine access. Incentives, featured in studies (n = 8), were utilized to motivate individuals toward vaccination. A telephone hotline (n = 2) was established, enabling individuals to seek information and clarification through telephone interviews, thereby fostering engagement and addressing queries related to influenza vaccination. Furthermore, champions and competitions, as observed in studies (n = 3), leveraged competitive dynamics and leadership roles to promote vaccination awareness. The use of emails and text messages (n = 3) emerged as effective tools for communication and reminders.

The educational interventions (n = 6), with a predominant focus on educational facets, have manifested as an ordinary constituent within the strategies under examination. Notably, other intervention categories also had an educational component by using different strategies such as printed materials like newsletters/papers, informative papers, posters, promotion materials (n = 8), and educational videos (n = 2). These interventions played a role in increasing awareness and knowledge pertaining to influenza vaccination achieved through the implementation of diverse information dissemination strategies tailored to the HCP audience. However, it should be noted that few studies found education ineffective in promoting vaccine attitudes or uptake (n = 3).

Mobile cart programs and mobile vaccination services were also examined as a solo intervention in some studies (n = 2) and as an important component in others (n = 13), with a particular emphasis on their relevance in healthcare settings. These programs were found to offer on-site vaccination services facilitated through mobile carts, thus ensuring convenient and readily accessible avenues for HCPs to receive vaccinations.

Additionally, the requirement of unvaccinated employees to wear masks (n = 4), and informed declaration strategies (n = 6) focused on ensuring that unvaccinated employees were well informed about the implications of their vaccination status and the potential consequences, fosters informed decision-making among HCPs and ensures high vaccination rates. These diverse intervention components collectively underscore the multifaceted strategies harnessed to promote influenza immunization.

As per the comparative efficacy of the interventions examined in the reviewed studies, The collective findings from studies examining the efficacy of educational interventions in increasing influenza vaccination rates among HCPs reveal a mixed picture. Some studies did not provide compelling evidence that the educational intervention significantly improved vaccination uptake among HCPs (n = 3). In another study (n = 1), those who viewed a free vaccine intervention favorably were likelier to opt for vaccination over an educational approach, suggesting a preference for tangible incentives. Overall, these studies collectively suggest that while education plays a role in vaccination promotion, it may not always yield significant improvements in HCP vaccination rates, and alternative strategies may be more effective in certain contexts. In comparing various intervention approaches, some clear trends emerged. A comprehensive approach incorporating combined interventions (n = 5) demonstrated the most favorable outcomes. This inference underscores that comprehensive strategies are likely to address a broader range of factors influencing the outcome, leading to improved overall effectiveness.

In summary, several interventions aimed at cultivating positive attitudes toward influenza vaccination among HCPs could be identified by this review: these primarily included campaigns, educational interventions, and mobile art programs. Campaigns employed multiple strategies, including providing free vaccines, incentives, telephone hotlines, champions, competitions, emails, and text messages, to increase vaccine coverage. Educational interventions focused on disseminating information through printed materials, posters, educational videos, and newsletters, although some studies found education to be ineffective. Mobile cart programs and mobile vaccination services offered on-site vaccination services through mobile carts. Comprehensive approaches that combined interventions were found to be the most effective at improving vaccination rates, emphasizing the importance of multifaceted strategies in promoting influenza immunization among HCPs.

#### 3.2.4. Pandemic Overlap: Intersecting Attitudes toward Influenza and COVID-19 Vaccines

In light of the current global context, this review provided insights into how the COVID-19 pandemic influenced healthcare providers’ attitudes toward influenza vaccination. The findings demonstrated that the dynamic interaction between these two infectious diseases had a profound effect on HCPs’ choices regarding the uptake of influenza vaccination. Specifically, there was a noticeable increase in vaccination rates once the pandemic had commenced (n = 7). A significant portion of HCPs acknowledged the importance of receiving the influenza vaccine, recognizing its role in curbing influenza cases in the healthcare system. Furthermore, it played a crucial role in distinguishing, lessening, and managing symptoms that overlapped between COVID-19 and influenza (n = 2).

Interestingly, nurses exhibited the highest vaccination rate during the 2020–2021 period compared to other HCPs, with approximately 33.5% of nurses opting for influenza vaccination (n = 1). Furthermore, the review established a connection between individuals’ intention to receive the influenza vaccine and several COVID-19-related factors. These factors included experiencing and the fear of experiencing physical exhaustion due to COVID-19, feeling exhausted due to other COVID-19 protective measures, and reporting few side effects from the COVID-19 vaccination (n = 1). Moreover, the review shed light on how crisis management strategies employed during the COVID-19 pandemic led to a diminished perception of the necessity of influenza vaccine, particularly in certain regions like Saudi Arabia, resulting in lower rates of uptake (n = 1).

**Figure 4:**
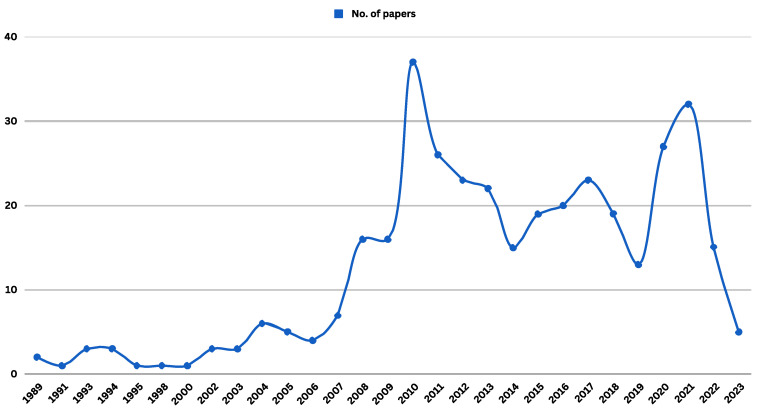
Timeline distribution of articles published on healthcare providers’ attitudes toward influenza vaccination.

The graph presented in Figure 4 exhibits two prominent peaks in the quantity of research studies. The initial peak occurred in 2010, coinciding with the outbreak of the H1N1 pandemic, while the subsequent peak emerged in 2021, following the onset of the COVID-19 pandemic. This pattern underscores the tendency for intensified research endeavors worldwide in the aftermath of pandemic occurrences, followed by a subsequent decline in research activity. Consequently, it is imperative to advocate for a global initiative aimed at sustaining research efforts and enhancing awareness campaigns among healthcare professionals. This proactive approach is vital for preparedness in the face of potential pandemics, as opposed to reactive measures to mitigate their effects post-occurrence.

#### 3.2.5. Twin Challenges: Mandatory Policy Impact on Attitudes and Influenza Vaccination

This review uncovered a noteworthy discourse surrounding mandatory vaccination policies and their influence on HCPs’ attitudes. Considerable disparities exist in the rate of support for or acceptance of mandatory vaccination policies within healthcare settings. A range of studies presented figures spanning from as low as 10–35.7%, indicating a significant variation in favor of such policies (n = 7). In contrast, a prevailing majority of rates, found in multiple studies, fall within the range of 46–76.5%, underlining a substantial level of acceptance and endorsement of mandatory vaccination (n = 12). And yet still a small subset of studies reported even higher rates, with figures ranging from 85% to 90.5% in support for mandatory vaccination policies, signifying robust support among certain populations (n = 4).

On the contrary, rates of opposition to mandatory vaccination policies ranged from 9% to 17.4% in select papers (n = 3). Meanwhile, another segment of rates, situated between 36% and 61.3%, indicates a notable degree of resistance to or non-acceptance of such policies in various contexts (n = 5). This array of statistics underscores the divergent attitudes and opinions surrounding the issue of mandatory vaccination within healthcare provider populations.

Notably, this comprehensive review also brought to light an expected correlation: HCPs who advocate for the implementation of mandatory influenza vaccination were found to be significantly more inclined to receive the influenza vaccine themselves (n = 9). Moreover, when examining the dynamics among different HCPs, a study concluded that physicians, in particular, demonstrated a stronger consensus on the assertion that ‘HCPs have a professional duty to undergo vaccination’. Additionally, a notable agreement emerged within this subgroup, asserting that ‘if all other options have been exhausted, legislation should mandate universal vaccination for HCPs during a pandemic influenza outbreak,’ surpassing the level of agreement among nurses (n = 1). However, it is worth noting that another study yielded a distinct perspective, suggesting that both physicians and nurses exhibited a high degree of willingness to receive the influenza vaccine if they were informed of its alignment with national healthcare policy, with 72.8% expressing readiness to vaccinate (n = 1). Furthermore, within the realm of mandatory vaccination, physicians emerged as the group most amenable to such a policy, particularly when it was offered directly within their workplace (n = 1). This observation underscores the nuanced variations in healthcare provider attitudes toward vaccination and the multifaceted nature of individuals’ considerations.

There exists a multitude of factors that foster a positive disposition toward mandatory influenza vaccination among HCPs, each contributing to the overall encouragement of such policies. Key elements encompass the provision of comprehensive information about the vaccine’s safety and efficacy (n = 2). the intrinsic motivation to safeguard patients’ well-being by being vaccinated (n = 3), the perception of influenza vaccination as an effective preventive measure (n = 2), the availability of the vaccine free of charge for caregivers of the elderly (n = 1), the convenience of vaccine access within the workplace setting (n = 3), and the implementation of a requirement for HCPs to sign a written declination form (n = 3). Conversely, factors that impede the acceptance of mandatory influenza vaccination policies are rooted in concerns over personal freedom and autonomy infringement (n = 2), apprehensions regarding potential vaccine side effects (n = 1), and reservations regarding the vaccine’s effectiveness in mitigating influenza (n = 1). These opposing influences highlight the intricate balance that HCPs navigate when forming their attitudes toward mandatory influenza vaccination, reflecting a complex interplay of personal, ethical, and practical considerations.

In summary, this review revealed significant disparities in support for mandatory vaccination policies, with a notable correlation between those advocating for mandatory vaccination and their willingness to receive the influenza vaccine themselves. Physicians tend to show a stronger consensus on the professional duty of HCPs to undergo vaccination. Factors fostering a positive disposition toward mandatory vaccination include comprehensive vaccine information, patient well-being motivation, perceived vaccine effectiveness, free vaccine availability for caregivers, workplace convenience, and declination form implementation. Conversely, concerns over personal freedom, vaccine side effects, and doubts about vaccine effectiveness hinder acceptance of mandatory vaccination policies. These findings underscore the complex and multifaceted nature of HCPs’ attitudes toward mandatory influenza vaccination.

## 4. Discussion

The attitudes of HCPs toward influenza vaccines are shaped by several interconnected factors. Their motivations for vaccination encompass personal, patient, and family protection, with a strong belief in vaccine effectiveness as a driving force for vaccination uptake. This alignment of motivations demonstrates a dual commitment to personal well-being and public health. Conversely, barriers to vaccination among HCPs are rooted in concerns such as fear of side effects, doubts about vaccine efficacy, time constraints, and a perception of low personal risk. Some providers question the necessity of vaccination, viewing influenza as a non-serious illness. These barriers need to be effectively addressed to improve vaccination rates. Demographics also play a role in HCPs’ vaccination decisions. Age, gender, and professional responsibilities all influence choices. Recognizing these demographic variations is crucial for targeted outreach and communication/vaccine campaign strategies. Furthermore, organizational factors within healthcare institutions significantly impact vaccination rates. Factors like peer support, workplace culture, and institutional policies can either facilitate or hinder vaccination. Access to free vaccines, vaccination campaigns, and leadership recommendations are instrumental in encouraging vaccination. Therefore, creating a supportive vaccination culture within the healthcare approach seems to be effective for increasing vaccine uptake among providers [25,26].

Although mandatory vaccination policies seem to be effective in increasing vaccination rates in both HCPs and the general public, multifaceted approaches integrating education materials, behavior change, and policies would likely be more beneficial so that barriers could be addressed and hesitancy could be decreased. Maltezou et al. [34] asserted that while mandatory vaccinations can be beneficial for protecting healthcare workers from vaccine-preventable diseases, their success hinges on addressing factors such as vaccine hesitancy, mistrust, and misconceptions among healthcare workers. Consistent with this perspective, our review revealed that multifaceted interventions have demonstrated effectiveness in improving vaccination rates among HCPs. Instead of relying on a single strategy, these interventions combine various approaches to address the complex factors influencing vaccination decisions. Such multifaceted strategies might include a combination of educational programs, vaccination campaigns, free vaccine access, incentives, mandatory policies, and leadership recommendations.

Besides comprehensiveness, flexibility and adaptability within strategies are indeed vital. Healthcare settings can differ significantly, from large hospitals to small clinics, and healthcare provider subgroups within these settings may have unique needs and concerns. Therefore, interventions must be tailored to the specific context, taking into account the local culture, resources, and the characteristics of the healthcare workforce. The World Health Organization [35] emphasizes the critical importance of tailoring vaccination programs for success. While this process may require time, it ultimately ensures that interventions are not only effective but also represent a cost-effective investment in public health.

### 4.1. Prospects for Future Research

Our findings provide a roadmap for future research to refine strategies for promoting positive attitudes, addressing hesitancy, and optimizing interventions among this critical population. This collective understanding is pivotal for the formulation of evidence-based policies and interventions to enhance influenza vaccination rates among HCPs and, consequently, the broader community. Building on the identified factors that promote positive attitudes, future endeavors can focus on the design and implementation of targeted interventions to harness the facilitators identified. Investigating the most effective strategies for raising awareness, imparting education, and fostering a sense of professional responsibility among HCPs can yield practical guidelines, especially taking into account intrinsic and extrinsic motivation. Further research can also examine HCPs’ distinct attitudes toward different influenza variants. Investigating the reasons behind varying perspectives on seasonal strains, variants, and emerging strains can contribute to tailored communication strategies and interventions. Future studies can also explore the ethical, legal, and practical dimensions of implementing mandatory vaccination policies, considering both potential benefits and challenges.

### 4.2. Limitations

Our eligibility criteria focused on HCPs and their attitudes toward influenza vaccination. However, the definitions of HCPs and attitudes varied across studies, which could impact the consistency and comparability of findings. Further, the review process involved a considerable number of studies that were excluded at various stages due to eligibility criteria. While this exclusion process was conducted meticulously, there is potential for subjectivity in decision-making, albeit mitigated by the dual review process. Finally, the scope of the study focused primarily on the published literature in English and may not capture relevant studies in other languages or grey literature, which could introduce language and publication bias.

## 5. Conclusions

This comprehensive systematic review offers valuable insights into HCPs’ attitudes toward influenza vaccination. By charting the factors that foster positive attitudes while also identifying and understanding barriers, this review equips public health practitioners, researchers, and policymakers with essential knowledge. This, in turn, empowers them to design evidence-based programs and strategies aimed at increasing vaccination uptake among HCPs, whose attitudes and behaviors can significantly impact public perception. Therefore, ensuring that HCPs have the necessary tools and support to follow through on vaccination acceptance and uptake is of utmost importance. Given how many vaccination barriers identified in this review were systemic in nature, ensuring institutional commitment is garnered and the organizational culture and climate are assessed and addressed prior to and during vaccination interventions is critical to supporting HCPs’ uptake. By strengthening HCPs’ commitment to vaccination, we can enhance their ability to serve as effective advocates for vaccination within the healthcare system and the broader community, ultimately contributing to improved vaccination rates and public health outcomes.

## Figures and Tables

**Table 1:** Themes and Meanings.

Theme	Meaning
(1) Fostering Positive Views: Factors Promoting Attitudes toward Influenza Vaccination	▪ Personal and family protection ▪ Patient safety ▪ Transmission risk reduction ▪ Professional responsibility ▪ Knowledge ▪ Perception of vaccine efficacy ▪ Worksite recommendations ▪ Accessibility and convenience ▪ Risk perception and severity
(2) Navigating Hesitancy: Barriers and Challenges to Attitudes on Influenza Vaccination	▪ Fear of side effects ▪ Concerns related to vaccine efficacy ▪ Lack of time ▪ Perception of being healthy/not at risk ▪ Beliefs that vaccination is not necessary ▪ Beliefs that influenza is not a serious illness ▪ Disliking injections ▪ Fear of allergic reactions ▪ Cost and access ▪ Pregnancy or breastfeeding ▪ Having a chronic illness
(3) Empowering Change: Interventions and Their Impact on HCPs’ Attitudes	▪ Campaigns ▪ Educational interventions ▪ Mobile art programs ▪ Provision of free vaccines ▪ Incentives
(4) Pandemic Overlap: Intersecting Attitudes toward Influenza and COVID-19 Vaccination	▪ Increasing vaccination rates amid the pandemic ▪ Influenza vaccination as a way to distinguish COVID-19 cases ▪ Fear of experiencing physical exhaustion due to COVID-19 ▪ Feeling exhausted due to COVID-19 protective measures
(5) Twin Challenges: Mandatory Policy Impact on Attitudes and Influenza Vaccination	▪ HCPs who support mandatory influenza vaccination are more likely to be vaccinated against influenza ▪ Physicians tend to be the most supportive group of mandatory vaccination policies ▪ Several intrinsic and extrinsic factors impact mandatory vaccination policies

## Data Availability

All data were provided as supplementary documents to this article.

## Supplementary Materials

The following supporting information can be downloaded at: https://www.mdpi.com/article/10.3390/vaccines11121760/s1, Supplementary document S1: Search strategy for each database; Supplementary document S2: Complete characteristics and quality assessment of the included articles; Supplementary document S3: Full citations for the results section.
