# Supplementary material for: Flu Shots Unveiled: A Global Systematic Review of Healthcare Providers’ Uptake of, Perceptions, and Attitudes toward Influenza Vaccination
Source: Vaccines (Basel). 2023 Nov 27;11(12):1760. doi: 10.3390/vaccines11121760 (PMC10747442; doi:10.3390/vaccines11121760)
Supplement: Supplementary file 1 [file vaccines-11-01760-s001.zip › Supplementary document S1.pdf]

Supplementary Table S1. Search strategy for each database

Database	Search Strategy
PUBMED	((((Nurses[MeSH Terms]) OR (nurses, public health[MeSH Terms])) OR (nurses, community health[MeSH Terms])) OR ((((((physicians[MeSH Terms]) OR (health personnel[MeSH Terms])) OR ("medical assistants")) OR (physician assistants[MeSH Terms])) OR (community health workers[MeSH Terms])) OR (rural health personnel[MeSH Terms]))) AND ((influenza vaccine[MeSH Terms]) AND ((((((((((((((((((((((((((attitude[MeSH Terms]) OR (attitude of health personnel[MeSH Terms])) OR (attitude to vaccines[MeSH Terms])) OR (cultural bias[MeSH Terms])) OR ("cultural values")) OR (Family attitudes[MeSH Terms])) OR (parental attitudes[MeSH Terms])) OR (maternal attitudes[MeSH Terms])) OR (paternal attitudes[MeSH Terms])) OR (uncertainty[MeSH Terms])) OR (intention[MeSH Terms])) OR (vaccination hesitancy[MeSH Terms])) OR (vaccination refusal[MeSH Terms])) OR (challenges)) OR (facilitators)) OR ("knowledge attitudes and practices")) OR ("KAP's")) OR (stigma[MeSH Terms])) OR (health services accessibility[MeSH Terms])) OR ("personal boundaries")) OR (barriers)) OR (enable)) OR (health beliefs[MeSH Terms])) OR (attitude to health[MeSH Terms])) OR (cultural sensitivity[MeSH Terms])) OR ("(intervention* OR polic*) AND promot* AND attitude*")))
PsycINFO	(Nurses OR "public health nurses" OR "community health nurses" OR physicians OR "health personnel" OR "medical assistants" OR "physician assistants" OR "community health workers" OR "rural health personnel" OR "rural health workers") AND "influenza vaccine" AND (attitude OR "attitude of health personnel" OR "attitude to vaccines" OR "cultural bias" OR "cultural values" OR "Family attitudes" OR "parental attitudes" OR "maternal attitudes" OR "paternal attitudes" OR uncertainty OR intention OR "vaccination hesitancy" OR "vaccination refusal" OR challenges OR facilitators OR "knowledge attitudes and practices" OR "KAP's" OR stigma OR "health services accessibility" OR "personal boundaries" OR barriers OR enable OR "health belief" OR "attitude to health" OR "cultural sensitivity" OR intervention* OR policy OR policies OR promot*)

<table>
<tr><td>CINAHL</td><td>
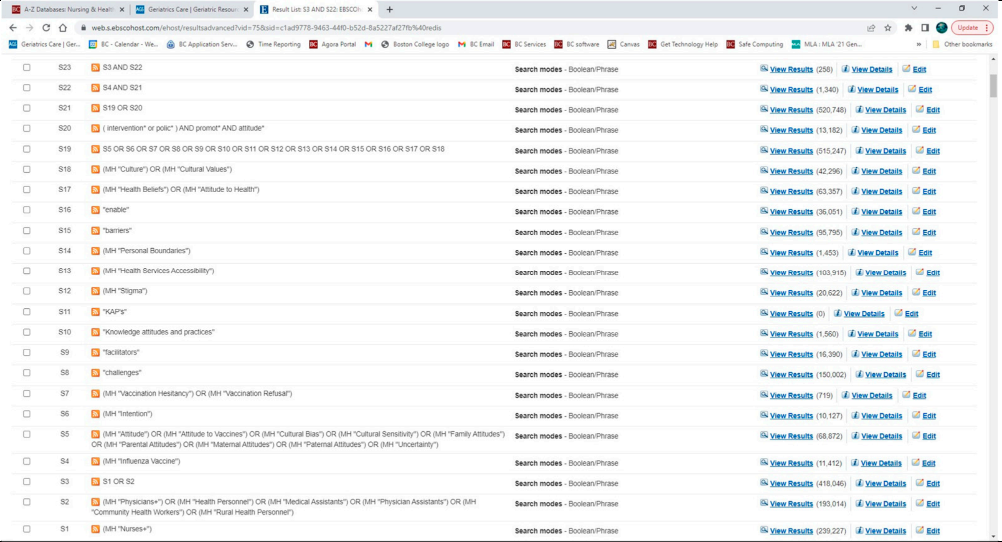
<table>
<tr><td>S23</td><td>S3 AND S22</td><td>Search modes - Boolean/Phrase</td><td>View Results (258) View Details Edit</td></tr>
<tr><td>S22</td><td>S4 AND S21</td><td>Search modes - Boolean/Phrase</td><td>View Results (1,340) View Details Edit</td></tr>
<tr><td>S21</td><td>S19 OR S20</td><td>Search modes - Boolean/Phrase</td><td>View Results (520,748) View Details Edit</td></tr>
<tr><td>S20</td><td>(intervention* or polic*) AND promot* AND attitude*</td><td>Search modes - Boolean/Phrase</td><td>View Results (13,182) View Details Edit</td></tr>
<tr><td>S19</td><td>S5 OR S6 OR S7 OR S8 OR S9 OR S10 OR S11 OR S12 OR S13 OR S14 OR S15 OR S16 OR S17 OR S18</td><td>Search modes - Boolean/Phrase</td><td>View Results (515,247) View Details Edit</td></tr>
<tr><td>S18</td><td>(MH "Culture") OR (MH "Cultural Values")</td><td>Search modes - Boolean/Phrase</td><td>View Results (42,296) View Details Edit</td></tr>
<tr><td>S17</td><td>(MH "Health Beliefs") OR (MH "Attitude to Health")</td><td>Search modes - Boolean/Phrase</td><td>View Results (63,357) View Details Edit</td></tr>
<tr><td>S16</td><td>"enable"</td><td>Search modes - Boolean/Phrase</td><td>View Results (36,051) View Details Edit</td></tr>
<tr><td>S15</td><td>"barriers"</td><td>Search modes - Boolean/Phrase</td><td>View Results (95,795) View Details Edit</td></tr>
<tr><td>S14</td><td>(MH "Personal Boundaries")</td><td>Search modes - Boolean/Phrase</td><td>View Results (1,453) View Details Edit</td></tr>
<tr><td>S13</td><td>(MH "Health Services Accessibility")</td><td>Search modes - Boolean/Phrase</td><td>View Results (103,915) View Details Edit</td></tr>
<tr><td>S12</td><td>(MH "Stigma")</td><td>Search modes - Boolean/Phrase</td><td>View Results (20,622) View Details Edit</td></tr>
<tr><td>S11</td><td>"KAP's"</td><td>Search modes - Boolean/Phrase</td><td>View Results (0) View Details Edit</td></tr>
<tr><td>S10</td><td>"Knowledge attitudes and practices"</td><td>Search modes - Boolean/Phrase</td><td>View Results (1,560) View Details Edit</td></tr>
<tr><td>S9</td><td>"facilitators"</td><td>Search modes - Boolean/Phrase</td><td>View Results (16,390) View Details Edit</td></tr>
<tr><td>S8</td><td>"challenges"</td><td>Search modes - Boolean/Phrase</td><td>View Results (150,002) View Details Edit</td></tr>
<tr><td>S7</td><td>(MH "Vaccination Hesitancy") OR (MH "Vaccination Refusal")</td><td>Search modes - Boolean/Phrase</td><td>View Results (719) View Details Edit</td></tr>
<tr><td>S6</td><td>(MH "Intention")</td><td>Search modes - Boolean/Phrase</td><td>View Results (10,127) View Details Edit</td></tr>
<tr><td>S5</td><td>(MH "Attitude") OR (MH "Attitude to Vaccines") OR (MH "Cultural Bias") OR (MH "Cultural Sensitivity") OR (MH "Family Attitudes") OR (MH "Parental Attitudes") OR (MH "Maternal Attitudes") OR (MH "Paternal Attitudes") OR (MH "Uncertainty")</td><td>Search modes - Boolean/Phrase</td><td>View Results (68,872) View Details Edit</td></tr>
<tr><td>S4</td><td>(MH "Influenza Vaccine")</td><td>Search modes - Boolean/Phrase</td><td>View Results (11,412) View Details Edit</td></tr>
<tr><td>S3</td><td>S1 OR S2</td><td>Search modes - Boolean/Phrase</td><td>View Results (418,046) View Details Edit</td></tr>
<tr><td>S2</td><td>(MH "Physicians+") OR (MH "Health Personnel") OR (MH "Medical Assistants") OR (MH "Physician Assistants") OR (MH "Community Health Workers") OR (MH "Rural Health Personnel")</td><td>Search modes - Boolean/Phrase</td><td>View Results (193,014) View Details Edit</td></tr>
<tr><td>S1</td><td>(MH "Nurses+")</td><td>Search modes - Boolean/Phrase</td><td>View Results (239,227) View Details Edit</td></tr>
</table>
</td></tr>
<tr><td>EMBASE</td><td>(((((Nurses OR (nurses, public health]) OR (nurses, community health)) OR ((physicians) OR (health personnel) OR (medical assistants) OR (physician assistant)) OR ((community health workers[) OR (rural health personnel[))) AND ((influenza vaccine)) AND (((attitudes) OR (health personnel attitude) OR (attitude to vaccines)) OR ((cultural bias) OR (cultural values)) OR (Family attitudes) OR (parental attitudes) OR (maternal attitudes[) OR (paternal attitudes)) OR ((uncertainty) OR (vaccination hesitancy) OR (vaccination refusal)) OR ((challenges) OR (facilitators)) OR (("knowledge attitudes and practices") OR ("KAP's")) OR ((stigma)) OR ((health services accessibility)) OR (("personal boundaries") OR (barriers)) OR (enable)) OR ((health beliefs) OR (attitude to health) OR (cultural sensitivity)) OR ((("intervention* OR polic*))) AND (promot* AND attitude*")))))/br</td></tr>
<tr><td>SCOPUS</td><td>All Fields “influenza vaccine” AND All Fields “attitude* OR "cultural bias" OR "cultural values" OR uncertainty OR intention OR "vaccination hesitancy" OR "vaccination refusal" OR challenges OR facilitators OR "knowledge attitudes and practices" OR kap's OR stigma OR "health services accessibility" OR</td></tr>
</table>

	"personal boundar*" OR barrier* OR enable OR "health beliefs" OR "cultural sensitivity" OR "(intervention* OR polic*) AND promot* AND attitude*" AND<br><br>All Fields nurs* OR physician* OR "health* personnel" OR "rural health" OR "Community health"<br><br>NOTE: When I searched "influenza vaccine* (Keyword) AND attitude* etc in Keyword field,result was 0.<br>I had to combine all the terms with attitude under attitude* and had to use the field "Article Title, Abstract, Keywords.<br>Then I used the broad terms for health care personnel to go from 3802 results to 1813 final results.<br>Using Abstracts includes too many extraneous results. The SCOPUS & Web of Science Search Engines are not perfect.
Web of Science	nurse* OR physician* OR "physician assistant*" OR "healthcare personnel" OR "health care personnel*" OR "rural health worker*" OR "medical assistant*"<br><br>AND "influenza vaccine"<br><br>AND attitude OR "attitude of health personnel" OR "attitude to vaccines" OR "cultural bias" OR "cultural values" OR "Family attitudes" OR uncertainty OR intention OR "vaccination hesitancy" OR "vaccination refusal" OR facilitators OR "knowledge attitudes and practices" OR "KAP's" OR stigma OR "health services accessibility" OR "personal boundaries" OR barriers OR enable OR "health beliefs" OR "attitude to health" OR "cultural sensitivity" OR intervention* OR policy OR policies OR promot*

Notes:

PsycINFO Citations dated back to 1937
CINAHL online dated back to 1937
PsycInfo online dates back to 1840.
EMBASE online dates back to 1966
PubMed Online dates back to 1785 and older. Our search dated back to 1981.
Web of Science online dates back to 1900
SCOPUS online dates back to 1944. Our search dated back to 1979.
