# Supplementary material for: Flu Shots Unveiled: A Global Systematic Review of Healthcare Providers’ Uptake of, Perceptions, and Attitudes toward Influenza Vaccination
Source: Vaccines (Basel). 2023 Nov 27;11(12):1760. doi: 10.3390/vaccines11121760 (PMC10747442; doi:10.3390/vaccines11121760)
Supplement: Supplementary file 1 [file vaccines-11-01760-s001.zip › Supplementary document S3.pdf]

1. Fostering Positive Views: Factors Promoting Attitudes towards Influenza Vaccines

The review illuminated multifaceted factors that act as catalysts in fostering positive attitudes among healthcare providers towards influenza vaccines. These encompassed an assortment of intrinsic and extrinsic motivators that collectively highlight the motivations, perceptions, and concerns affecting healthcare providers' decisions to receive flu vaccination. It was noted that healthcare providers' motivations for receiving influenza vaccinations are consistently centered around self-protection (with reported rates ranging from 53.4% to 87%), patient protection (with reported rates ranging from 31% to 63%), and family protection. These reasons are frequently cited across various studies [1-272].

The review also revealed that healthcare providers are motivated by concerns about transmitting influenza specifically to vulnerable patients (with reported rates ranging 21% to 36%), their families, and themselves. Additionally, healthcare providers' belief in the vaccine's effectiveness and its role in preventing the spread of influenza, minimizing viral reservoir, and reducing hospital visits significantly influences their decision to get vaccinated [1, 2, 3, 4, 5, 6, 7, 8, 9, 10, 11, 12, 13, 14, 15, 16, 17, 18, 19, 20, 21, 22, 23, 24, 25, 26, 27, 28, 29, 30, 31, 32, 33, 34, 35, 36, 37, 38, 39, 40, 41, 42, 43, 44, 45, 46, 48, 49, 50, 51, 52, 53, 54, 55, 56, 57, 58, 59, 60, 61, 62, 63, 64, 65, 66, 67, 68, 69, 70, 71, 72, 73, 74, 75, 76, 77, 78, 79, 80, 82, 83, 84, 85, 86, 88, 89, 90, 91, 92, 93, 94, 95, 96, 97, 98, 99, 100, 101, 102, 103, 104, 105, 106, 107, 108, 109, 110, 111, 112, 113, 114, 115, 116, 117, 118, 119, 120, 121, 122, 123, 124, 125, 126, 127, 128, 129, 130, 131, 132, 133, 134, 135, 136, 137, 138, 139, 140, 141, 142, 143, 144, 145, 146, 147, 148, 149, 150, 151, 152, 153, 154, 155, 156, 157, 158, 159, 160, 161, 162, 163, 164, 165, 166, 167,

168, 169, 170, 171, 172, 173, 174, 175, 176, 177, 178, 179, 180, 181, 182, 183, 184, 185, 186, 187, 188, 189, 190, 191, 192, 193, 194, 195, 196, 197, 199, 200, 201, 202, 203, 204, 206, 207, 208, 209, 210, 211, 212, 213, 214, 215, 216, 217, 218, 219, 220, 221, 222, 223, 224, 226, 227, 228, 229, 230, 231, 232, 233, 234, 235, 236, 237, 238, 239, 240, 241, 242, 243, 244, 245, 246, 247, 248, 249, 250, 251, 252, 253, 254, 255, 256, 257, 258, 259, 260, 261, 262, 263, 264, 265, 266, 267, 268, 269, 270, 271, 272, 273, 274, 275, 276]. Furthermore, factors such as being older, spending longer time in the profession, being vaccinated before, possessing correct knowledge about vaccine efficacy and safety (encompassing beliefs in proven vaccine efficacy and dispelling misconceptions, such as links between vaccines and autism), as well as professional responsibilities and the perception of vaccination as being a fundamental part of their obligation to ensure patient safety and maintain optimal healthcare delivery, together promoted healthcare providers' vaccination decisions [1, 2, 3, 4, 5, 6, 7, 8, 9, 10, 11, 12, 13, 14, 15, 16, 17, 18, 19, 20, 21, 22, 23, 24, 25, 26, 27, 28, 29, 30, 31, 32, 33, 34, 35, 36, 37, 38, 39, 40, 41, 42, 43, 44, 45, 46, 48, 49, 51, 52, 53, 54, 55, 56, 57, 58, 59, 60, 61, 62, 63, 64, 65, 66, 67, 68, 69, 70, 71, 72, 73, 74, 75, 76, 77, 78, 79, 80, 82, 83, 84, 85, 86, 88, 89, 90, 91, 92, 93, 94, 95, 96, 98, 99, 100, 101, 102, 104, 105, 106, 107, 108, 109, 110, 111, 112, 113, 114, 115, 116, 117, 118, 119, 120, 121, 122, 123, 124, 125, 126, 127, 128, 129, 130, 131, 132, 133, 134, 135, 136, 137, 138, 139, 140, 141, 142, 143, 144, 145, 146, 147, 148, 149, 150, 151, 152, 153, 154, 155, 156, 157, 158, 159, 160, 161, 162, 163, 164, 165, 166, 167, 168, 169, 170, 171, 172, 173, 174, 175, 176, 177, 178, 179, 180, 181, 182, 183, 184, 185, 186, 187, 188, 189, 190, 191, 192, 193, 194, 195, 196, 197, 199, 200, 201, 202, 203, 204, 206, 207, 208, 209, 210, 211, 212, 213, 214, 216, 217, 218, 219, 220, 221, 222, 223, 224, 226, 227, 228, 229, 230, 231, 232, 233, 234, 235, 236, 237, 238, 239, 240,

241, 242, 243, 244, 245, 246, 247, 248, 249, 250, 251, 252, 253, 254, 255, 256, 257, 258, 259, 260, 261, 262, 263, 264, 265, 266, 267, 268, 269, 270, 271, 272, 274, 277, 278].

At the level of healthcare settings, the presence of peer support, organizational culture, and institutional policies appeared to be significant in promoting vaccination among healthcare providers [1, 3, 4, 5, 6, 7, 8, 9, 10, 11, 12, 13, 14, 15, 16, 17, 18, 19, 20, 21, 22, 23, 24, 25, 26, 27, 28, 29, 30, 31, 32, 33, 34, 35, 36, 37, 38, 39, 40, 41, 42, 43, 44, 45, 46, 47, 48, 49, 51, 52, 53, 54, 55, 56, 57, 58, 59, 60, 61, 62, 63, 64, 65, 66, 67, 68, 69, 70, 71, 72, 73, 74, 75, 76, 77, 78, 79, 80, 82, 83, 84, 85, 86, 88, 89, 90, 91, 92, 93, 94, 95, 96, 97, 98, 99, 100, 101, 102, 104, 105, 106, 107, 108, 109, 110, 111, 112, 113, 114, 115, 116, 117, 118, 119, 120, 121, 123, 124, 125, 126, 127, 128, 129, 130, 131, 132, 133, 134, 135, 136, 137, 138, 139, 140, 141, 142, 143, 144, 145, 146, 147, 148, 149, 150, 151, 152, 153, 154, 155, 156, 157, 158, 159, 160, 161, 162, 163, 164, 165, 166, 167, 168, 169, 170, 171, 172, 173, 174, 175, 176, 177, 178, 179, 180, 181, 182, 183, 184, 185, 186, 187, 188, 189, 190, 191, 192, 193, 194, 195, 196, 197, 199, 200, 201, 202, 203, 204, 206, 207, 208, 209, 210, 211, 212, 213, 214, 216, 217, 218, 219, 220, 221, 222, 223, 224, 226, 227, 228, 229, 230, 231, 232, 233, 234, 235, 236, 237, 238, 239, 240, 241, 242, 243, 244, 245, 246, 247, 248, 249, 250, 251, 252, 253, 254, 255, 256, 257, 258, 259, 260, 261, 262, 263, 264, 265, 266, 267, 268, 269, 270, 271, 272, 279, 280]. Further, the availability of free vaccines and workplace access facilitated higher vaccination rates [2, 3, 5, 12, 22, 24, 25, 47, 50, 205, 281]. Workplace vaccination campaigns and recommendations by leaders [12, 24, 29], and specific training of vaccination campaigns [10, 282] also promoted uptake.

In summary, healthcare providers’ motivations for influenza vaccination encompass self-protection, patient protection, and family protection. The belief in vaccine effectiveness, along with concerns about transmitting influenza, also significantly influence their decision. Factors

like age, vaccination history, knowledge, and professional responsibilities contribute to their vaccination choices. Peer support, organizational culture, and institutional policies have a pivotal role in promoting vaccination within this group.

2. Navigating Hesitancy: Barriers and Challenges to Attitudes on Influenza Vaccines

Counterbalancing the positive aspects, this theme delved into the factors that serve as barriers to fostering favorable attitudes. Issues such as fear of side effects (rates ranged from 13% to 63%) [1, 3, 4, 7, 13, 14, 16, 17, 18, 19, 20, 24, 28, 33, 34, 35, 36, 38, 40, 44, 45, 48, 53, 54, 56, 60, 62, 63, 72, 76, 78, 79, 80, 82, 84, 85, 86, 92, 95, 96, 97, 100, 104, 105, 108, 109, 112, 116, 118, 119, 120, 121, 125, 132, 133, 135, 136, 137, 138, 139, 140, 142, 143, 144, 145, 147, 148, 150, 151, 153, 158, 162, 164, 166, 168, 171, 172, 175, 177, 178, 179, 180, 182, 183, 184, 185, 188, 193, 195, 200, 204, 205, 206, 208, 219, 220, 223, 224, 228, 229, 234, 235, 236, 238, 239, 241, 245, 246, 248, 250, 251, 252, 256, 257, 259, 260, 264, 265, 266, 268, 269, 271, 272, 274, 275, 279, 283, 284, 285, 286, 287, 288, 289, 290, 291, 292, 293, 294, 295, 296, 297, 298, 299, 300, 301, 302, 303, 304, 305, 306, 307, 308, 309, 310, 311, 312, 313, 314, 315, 316, 317, 318, 319, 320, 321, 322, 323, 324, 325, 326, 327, 328, 329, 330, 331], concerns related to vaccine efficacy (rates were ranging from 9% to 56%) [1, 2, 3, 4, 5, 7, 13, 17, 19, 20, 25, 28, 30, 33, 35, 38, 45, 50, 54, 55, 56, 60, 63, 70, 71, 72, 76, 78, 79, 80, 82, 83, 84, 85, 86, 88, 92, 96, 98, 100, 102, 109, 112, 113, 115, 116, 117, 118, 119, 120, 125, 129, 132, 133, 136, 138, 139, 140, 142, 143, 144, 146, 147, 148, 150, 151, 153, 158, 159, 161, 166, 168, 171, 175, 176, 177, 178, 179, 180, 183, 187, 188, 193, 206, 208, 210, 211, 214, 220, 224, 233, 234, 235, 245, 246, 250, 252, 256, 257, 259, 260, 262, 264, 265, 266, 268, 269, 271, 272, 279, 281, 282, 283, 284, 287, 288, 290, 292, 296, 298, 299, 300, 301, 302, 303, 304, 306, 308, 309, 310, 311, 312, 316, 317,

318, 319, 320, 321, 323, 324, 328, 330, 331, 332, 333, 334, 335, 336, 337, 338, 339, 340], lack of time to get vaccinated (rates were ranging from 22% to 47%) [7, 14, 18, 24, 40, 48, 54, 63, 71, 72, 78, 84, 85, 88, 94, 118, 120, 129, 136, 140, 146, 147, 148, 150, 151, 157, 162, 186, 187, 188, 193, 209, 233, 234, 238, 245, 246, 248, 252, 262, 269, 272, 282, 287, 290, 291, 292, 293, 302, 304, 309, 313, 314, 316, 318, 323, 324, 327, 330, 331, 333, 340, 341, 342], beliefs that they do not need to get the vaccine as they are healthy (rates were ranging from 9% to 35%) [5, 9, 14, 16, 45, 51, 63, 75, 78, 85, 88, 89, 97, 104, 105, 120, 122, 132, 150, 168, 178, 185, 214, 216, 284, 289, 303, 313, 341], perception of not being at risk/ being at low risk of contracting the infection (ranging from 2% to 32%) [7, 20, 25, 38, 41, 63, 71, 79, 86, 89, 110, 113, 116, 119, 120, 121, 124, 126, 132, 136, 139, 140, 145, 158, 172, 177, 179, 185, 195, 208, 209, 210, 222, 228, 234, 240, 246, 259, 262, 264, 266, 272, 288, 311, 315, 319, 321, 337, 338, 342, 343, 344], beliefs that vaccination is not necessary (ranging from 23% to 53%) [9, 13, 24, 55, 75, 84, 93, 96, 102, 144, 156, 187, 196, 202, 206, 223, 227, 235, 249, 285, 300, 320, 325, 345], beliefs that influenza is not a serious illness (ranging from 25% to 58%) [2, 18, 25, 54, 56, 66, 71, 100, 108, 112, 117, 132, 136, 139, 147, 151, 165, 170, 176, 183, 184, 195, 222, 241, 260, 275, 288, 295, 302, 304, 306, 312, 314, 315, 319, 336, 340, 344, 346], and beliefs that the vaccine causes illness (ranging from 16% to 38.5%) [5, 17, 25, 45, 51, 54, 60, 63, 92, 109, 112, 113, 118, 124, 129, 135, 136, 143, 146, 178, 202, 214, 219, 220, 238, 260, 265, 272, 281, 301, 302, 309, 315, 317, 323, 341, 347, 348, 349] were most commonly repeated in the literature as major contributors to vaccine hesitancy.

The review also revealed that healthcare providers are hindered from taking the vaccine because of disliking injections / fear of pain (ranging from 11% to 35%) [3, 7, 13, 18, 36, 45, 78, 80, 82, 85, 86, 88, 97, 113, 124, 132, 133, 136, 138, 140, 143, 144, 148, 153, 179, 183, 188, 197,

206, 222, 233, 234, 236, 238, 245, 246, 247, 260, 265, 269, 272, 293, 295, 298, 299, 302, 307, 309, 315, 319, 320, 323, 328, 329, 330, 331, 335, 336, 340, 343, 348, 350], and fear of local and systemic allergic reactions related to the vaccine [80, 97, 143, 164, 177, 179, 188, 214, 221, 224, 239, 245, 260, 297, 298, 301, 318, 332, 336, 343, 348].

System-related barriers including expensive costs [1, 3, 18, 80, 85, 148, 151, 171, 182, 183, 202, 221, 251, 256, 272, 279, 282, 286, 295, 314, 318, 319, 331, 333, 351, 352, 353], and lacking availability of the vaccine [13, 14, 40, 48, 51, 56, 72, 94, 100, 120, 121, 129, 147, 196, 197, 199, 214, 216, 233, 234, 247, 260, 262, 271, 287, 295, 302, 305, 313, 316, 318, 319, 324, 340, 347, 350, 351, 352] were consistently reported throughout the literature as well. Furthermore, demographic factors such as being younger and female sex were related to less uptake of the influenza vaccine [64, 70, 123, 129, 202, 204, 244, 354].

Also, having a current pregnancy or breastfeeding were reported as barriers to get the vaccine [15, 138, 143, 151, 224, 245, 255, 297, 313, 332] as well as having a chronic illness, having a medical contraindication, or lacking a medical indication for vaccination. [14, 34, 70, 72, 75, 76, 116, 139, 151, 153, 156, 168, 188, 260, 287, 291, 296, 299, 312, 332, 334, 339, 355, 356]. Some health care providers did not take the vaccine because of being against vaccination (i.e., anti-vaccination) in general. [9, 13, 14, 16, 108, 138, 157, 185, 265, 268, 282, 302, 304, 330].

In summary, several factors were identified as major impediments to fostering positive attitudes. These included concerns about vaccine side effects, vaccine efficacy, lack of time for vaccination, the belief that one is healthy and doesn't need the vaccine, the perception of being at low risk for infection, the belief that vaccination is unnecessary, and the fear that the vaccine itself could cause illness. Healthcare providers also faced barriers, such as a dislike of injections

or fear of pain, as well as concerns about allergic reactions related to the vaccine. System-related issues, like the cost and availability of the vaccine, were commonly cited obstacles. Demographic factors, such as younger age and female gender, were associated with lower vaccine uptake. Overall, these findings highlight a range of factors contributing to vaccine hesitancy in the context of influenza vaccination.

3. Empowering Change: Interventions and Their Impact on Healthcare Providers' Attitudes

The systematic analysis revealed a spectrum of interventions designed to cultivate positive attitudes [31, 68, 73, 88, 95, 96, 101, 105, 113, 129, 133, 143, 151, 156, 157, 164, 165, 168, 191, 224, 242, 260, 297, 298, 301, 304, 310, 311, 315, 317, 332, 339, 348, 350, 357, 358, 359, 360]. These interventions were categorized into three primary categories, each offering a unique approach employed to address the challenges of promoting influenza vaccination, cultivating positive attitudes, and increasing awareness of the value of influenza vaccination among health care providers. The categories included campaigns, educational interventions, and mobile art programs.

Tested vaccination **campaigns** [73, 88, 96, 101, 105, 113, 129, 143, 151, 156, 157, 164, 165, 191, 224, 242, 297, 304, 310, 311, 315, 317, 332, 339, 348, 350, 357, 358, 359, 360] were characterized by the application of multiple strategies aimed at bolstering vaccine coverage among the health care providers population. These strategies include the provision of free vaccines [73, 88, 96, 105, 113, 129, 143, 151, 224, 297, 304, 332, 358], effectively reducing financial barriers to vaccine access. Incentives, featured in studies [73, 96, 101, 113, 164, 242, 317, 332], were utilized to motivate individuals toward vaccination. A telephone hotline [224, 332] was established, enabling individuals to seek information and clarification through telephone

interviews, thereby fostering engagement and addressing queries related to influenza vaccination. Furthermore, champions and competitions, as observed in studies [73, 317, 348], leveraged competitive dynamics and leadership roles to promote vaccination awareness. The use of e-mails and text messages [88, 297, 317] emerged as effective tools for communication and reminders.

The educational interventions [31, 68, 133, 260, 298, 301], with a predominant focus on educational facets, have manifested as an ordinary constituent within the strategies under examination. Notably, other intervention categories also had an educational component by using different strategies such as printed materials like newsletters/papers, informative papers, posters, promotion materials [73, 88, 95, 101, 301, 304, 348, 358], and educational videos [129, 310]. These interventions played a role in increasing awareness and knowledge pertaining to influenza vaccination achieved through the implementation of diverse information dissemination strategies tailored to the health care providers audience. It should be noted though that few studies found education ineffective in promoting vaccine attitudes or uptake [73, 101, 191].

Mobile cart programs and mobile vaccination services were also examined as a solo intervention in some studies [95, 168] and as an important component in others [73, 88, 105, 113, 164, 242, 301, 304, 315, 317, 350, 357, 361], with a particular emphasis on their relevance in healthcare settings. These programs were found to offer on-site vaccination services facilitated through mobile carts, thus ensuring convenient and readily accessible avenues for healthcare providers to receive vaccinations.

Additionally, the requirement of unvaccinated employees to wear masks [156, 164, 339, 346], and informed declaration strategies [105, 113, 156, 167, 224, 315] focused on ensuring that unvaccinated employees were well informed about the implications of their vaccination status and the potential consequences, fostering informed decision-making among healthcare providers, and

ensuring high vaccination rates. These diverse intervention components collectively underscore the multifaceted strategies harnessed to promote influenza immunization.

As per the comparative efficacy of the interventions examined in the reviewed studies, The collective findings from studies examining the efficacy of educational interventions in increasing influenza vaccination rates among healthcare workers (HCWs) reveal a mixed picture. Some studies did not provide compelling evidence that the educational intervention significantly improved vaccination uptake among HCWs [73, 101, 129]. In another study [105], those who viewed a free vaccine intervention favorably were likelier to opt for vaccination over an educational approach, suggesting a preference for tangible incentives. Overall, these studies collectively suggest that while education plays a role in vaccination promotion, it may not always yield significant improvements in HCW vaccination rates, and alternative strategies may be more effective in certain contexts. In comparing various intervention approaches, some clear trends emerged. A comprehensive approach incorporating combined interventions [73, 101, 129, 151, 242] demonstrated the most favorable outcomes. This inference underscores that comprehensive strategies are likely to address a broader range of factors influencing the outcome, leading to improved overall effectiveness.

In summary, several interventions aimed at cultivating positive attitudes towards influenza vaccination among healthcare providers could be identified by this review: these primarily included campaigns, educational interventions, and mobile art programs. Campaigns employed multiple strategies, including providing free vaccines, incentives, telephone hotlines, champions, competitions, emails, and text messages, to increase vaccine coverage. Educational interventions focused on disseminating information through printed materials, posters, educational videos, and newsletters, although some studies found education to be ineffective.

Mobile cart programs and mobile vaccination services offered on-site vaccination services through mobile carts. Comprehensive approaches that combined interventions were found to be the most effective in improving vaccination rates, emphasizing the importance of multifaceted strategies in promoting influenza immunization among healthcare providers.

4. Pandemic Overlap: Intersecting Attitudes towards Influenza and COVID-19 Vaccines

In light of the current global context, this review provided insights on how the COVID-19 pandemic influenced healthcare provider's attitudes toward influenza vaccination. The findings demonstrated that the dynamic interaction between these two infectious diseases had a profound effect on healthcare providers' choices regarding uptake of influenza vaccination. Specifically, there was a noticeable increase in vaccination rates once the pandemic had commenced [2, 181, 230, 231, 321, 323, 362]. A significant portion of healthcare providers acknowledged the importance of receiving the influenza vaccine, recognizing its role in curbing influenza cases in the healthcare system. Furthermore, it played a crucial role in distinguishing, lessening and managing symptoms that overlapped between COVID-19 and influenza [231, 321].

Interestingly, nurses exhibited the highest vaccination rate during the 2020 to 2021 period compared to other healthcare providers, with approximately 33.5% of nurses opting for influenza vaccination [362]. Furthermore, the review established a connection between individuals' intention to receive the influenza vaccine and several COVID-19-related factors. These factors included experiencing and fear of experiencing physical exhaustion due to COVID-19, feeling exhausted due to other COVID-19 protective measures, and reporting few side effects from COVID-19 vaccination [201]. Moreover, the review shed light on how crisis management

strategies employed during the COVID-19 pandemic led to a diminished perception for the necessity of influenza vaccine, particularly in certain regions like Saudi Arabia, resulting in lower rates of uptake [363].

5. Twin Challenges: Mandatory Policy Impact on Attitudes and Influenza Vaccination

This review uncovered a noteworthy discourse surrounding mandatory vaccination policies and their influence on healthcare providers' attitudes. Considerable disparities exist in the rate of support for or acceptance of mandatory vaccination policies within healthcare settings. A range of studies presented figures spanning from as low as 10% to 35.7%, indicating a significant variation in favor of such policies [15, 62, 96, 231, 232, 341, 364]. In contrast, a prevailing majority of rates, found in multiple studies, fall within the range of 46% to 76.5%, underlining a substantial level of acceptance and endorsement of mandatory vaccination [25, 62, 98, 140, 174, 199, 233, 258, 261, 282, 323, 365]. And still yet, a small subset of studies reported even higher rates, with figures ranging from 85% to 90.5% in support for mandatory vaccination policies, signifying robust support among certain populations [67, 71, 90, 194].

On the contrary, rates of opposition to mandatory vaccination policies, ranged from 9% to 17.4% in select papers [214, 258, 261]. Meanwhile, another segment of rates, situated between 36% and 61.3%, indicates a notable degree of resistance to or non-acceptance of such policies in various contexts [15, 178, 232, 341, 366]. This array of statistics underscores the divergent attitudes and opinions surrounding the issue of mandatory vaccination within healthcare provider populations.

Notably, this comprehensive review also brought to light an expected correlation: healthcare providers who advocate for the implementation of mandatory influenza vaccination

were found to be significantly more inclined to receive the influenza vaccine themselves [20, 61, 155, 192, 207, 328, 341, 365, 367]. Moreover, when examining the dynamics among different healthcare providers, a study concluded that physicians, in particular, demonstrated a stronger consensus on the assertion that 'healthcare providers have a professional duty to undergo vaccination'. Additionally, a notable agreement emerged within this subgroup, asserting that 'if all other options have been exhausted, legislation should mandate universal vaccination for healthcare providers during a pandemic influenza outbreak,' surpassing the level of agreement among nurses [74]. However, it is worth noting that another study yielded a distinct perspective, suggesting that both physicians and nurses exhibited a high degree of willingness to receive the influenza vaccine if they were informed of its alignment with national healthcare policy, with a 72.8% expressing readiness to vaccinate [326]. Furthermore, within the realm of mandatory vaccination, physicians emerged as the group most amenable to such a policy, particularly when it was offered directly within their workplace [329]. This observation underscores the nuanced variations in healthcare provider attitudes towards vaccination and the multifaceted nature of individuals' considerations.

There exists a multitude of factors that foster a positive disposition towards mandatory influenza vaccination among healthcare providers, each contributing to the overall encouragement of such policies. Key elements encompass the provision of comprehensive information about the vaccine's safety and efficacy [48, 127], the intrinsic motivation to safeguard patients' well-being by getting vaccinated [67, 140, 258], the perception of influenza vaccination as an effective preventive measure [174, 365], the availability of the vaccine free of charge for caregivers of the elderly [127], the convenience of vaccine access within the workplace setting [48, 127, 329], and the implementation of a requirement for healthcare

workers to sign a written declination form [23, 195, 368]. Conversely, factors that impede the acceptance of mandatory influenza vaccination policies are rooted in concerns over personal freedom and autonomy infringement [178, 199], apprehensions regarding potential vaccine side effects [345], and reservations regarding the vaccine's effectiveness in mitigating influenza [176]. These opposing influences highlight the intricate balance that healthcare providers navigate when forming their attitudes toward mandatory influenza vaccination, reflecting a complex interplay of personal, ethical, and practical considerations.

In summary, this review revealed significant disparities in support for mandatory vaccination policies, with a notable correlation between those advocating for mandatory vaccination and their willingness to receive the influenza vaccine themselves. Physicians tend to show a stronger consensus on the professional duty of healthcare providers to undergo vaccination. Factors fostering a positive disposition towards mandatory vaccination include comprehensive vaccine information, patient well-being motivation, vaccine effectiveness perception, free vaccine availability for caregivers, workplace convenience, and declination form implementation. Conversely, concerns over personal freedom, vaccine side effects, and doubts about vaccine effectiveness hinder acceptance of mandatory vaccination policies. These findings underscore the complex and multifaceted nature of healthcare providers' attitudes towards mandatory influenza vaccination.

References

1	Hakim SA, Amin W, Allam MF, Fathy AM, Mohsen A. Attitudes, beliefs and practice of Egyptian healthcare workers towards seasonal influenza vaccination. Influenza Other Respir Viruses. 2021;15(6):778-788. doi:10.1111/irv.12868
2	Papageorgiou C, Mazeri S, Karaiskakis M, et al. Exploring vaccination coverage and attitudes of health care workers towards influenza vaccine in Cyprus. Vaccine. 2022;40(12):1775-1782. doi:10.1016/j.vaccine.2022.02.020
3	Youssef D, Berry A, Youssef J, Abou-Abbas L. Vaccination against influenza among Lebanese health care workers in the era of coronavirus disease 2019. BMC Public Health. 2022;22(1):120. Published 2022 Jan 18. doi:10.1186/s12889-022-12501-9
4	Tomljenovic M, Petrovic G, Antoljak N, Hansen L. Vaccination attitudes, beliefs and behaviours among primary health care workers in northern Croatia. Vaccine. 2021;39(4):738-745. doi:10.1016/j.vaccine.2020.11.049
5	Halpin C, Reid B. Attitudes and beliefs of healthcare workers about influenza vaccination. Nurs Older People. 2019;31(2):32-39. doi:10.7748/nop.2019.e1154
6	Bardenheier BH, Lindley MC, Ball SW, de Perio MA, Laney S, Gravenstein S. Cluster Analysis: Vaccination Attitudes and Beliefs of Healthcare Personnel. Am J Health Behav. 2020;44(3):302-312. doi:10.5993/AJHB.44.3.3
7	Jędrzejek MJ, Mastalerz-Migas A. Influenza Vaccination Coverage, Motivators for, and Barriers to Influenza Vaccination among Healthcare Workers in Wroclaw, Poland. Int J Environ Res Public Health. 2022;19(3):1586. Published 2022 Jan 30. doi:10.3390/ijerph19031586

8	Wilson R, Zaytseva A, Bocquier A, et al. Vaccine hesitancy and self-vaccination behaviors among nurses in southeastern France. Vaccine. 2020;38(5):1144-1151. doi:10.1016/j.vaccine.2019.11.018
9	Korkmaz N, Nazik S, Gümüştakım RŞ, et al. Influenza vaccination rates, knowledge, attitudes and behaviours of healthcare workers in Turkey: A multicentre study. Int J Clin Pract. 2021;75(1):e13659. doi:10.1111/ijcp.13659
10	Wilson R, Scronias D, Zaytseva A, et al. Seasonal influenza self-vaccination behaviours and attitudes among nurses in Southeastern France. Hum Vaccin Immunother. 2019;15(10):2423-2433. doi:10.1080/21645515.2019.1587274
11	Alharbi N, Almutiri A, Alotaibi F, Ismail A. Knowledge and healthcare professionals' perceptions of influenza vaccination in the Qassim region, Saudi Arabia (2019-2020). Hum Vaccin Immunother. 2021;17(5):1426-1431. doi:10.1080/21645515.2020.1820809
12	Štěpánek L, Nakládalová M, Vildová H, Boriková A, Janošíková M, Ivanová K. Demand and motivation for influenza vaccination among healthcare workers before and during the COVID-19 era: a cross-sectional survey. Hum Vaccin Immunother. 2021;17(9):3113-3118. doi:10.1080/21645515.2021.1911212
13	Awadalla NJ, Al-Musa HM, Al-Musa KM, et al. Seasonal influenza vaccination among primary health care workers in Southwestern Saudi Arabia. Hum Vaccin Immunother. 2020;16(2):321-326. doi:10.1080/21645515.2019.1666500
14	Ochoa-Hein E, Gutiérrez-López EN, Torres-Erazo DS, et al. Factors associated with influenza vaccination acceptance in Mexican healthcare workers: A multicenter cross-sectional study. Prev Med. 2021;148:106560. doi:10.1016/j.ypmed.2021.106560
15	Moretti F, Visentin D, Bovolenta E, et al. Attitudes of Nursing Home Staff Towards Influenza Vaccination: Opinions and Factors Influencing Hesitancy. Int J Environ Res Public Health. 2020;17(6):1851. Published 2020 Mar 12. doi:10.3390/ijerph17061851
16	Lee PH, Cowling BJ, Yang L. Seasonal influenza vaccination among Chinese health care workers. Am J Infect Control. 2017;45(5):575-578. doi:10.1016/j.ajic.2016.05.038
17	Flanagan P, Dowling M, Gethin G. Barriers and facilitators to seasonal influenza vaccination uptake among nurses: A mixed methods study. J Adv Nurs. 2020;76(7):1746-1764. doi:10.1111/jan.14360
18	Nguyen TTM, Lafond KE, Nguyen TX, et al. Acceptability of seasonal influenza vaccines among health care workers in Vietnam in 2017. Vaccine. 2020;38(8):2045-2050. doi:10.1016/j.vaccine.2019.12.047
19	Wang To K, Lee S, Lee SS. Intention of nurses to receiving influenza vaccination before the 2013-14 season. Hum Vaccin Immunother. 2015;11(6):1345-1350. doi:10.1080/21645515.2015.1034916
20	Cherif I, Kharroubi G, Bouabid L, et al. Knowledge, attitudes and uptake related to influenza vaccine among healthcare workers during the 2018-2019 influenza season in Tunisia. BMC Public Health. 2021;21(1):907. Published 2021 May 13. doi:10.1186/s12889-021-10970-y
21	Daugherty JD, Blake SC, Grosholz JM, Omer SB, Polivka-West L, Howard DH. Influenza vaccination rates and beliefs about vaccination among nursing home employees. Am J Infect Control. 2015;43(2):100-106. doi:10.1016/j.ajic.2014.08.021
22	Ramadhani BP, Soeroto AY, Suryadinata H, Rakhmilla LE. Nursing knowledge, attitude, and practice to influenza vaccination at suburban hospital in West Java, Indonesia. J Prev Med Hyg. 2020;61(1):E15-E20. Published 2020 Apr 2. doi:10.15167/2421-4248/jpmh2020.61.1.1119

23	Pless A, Shaw D, McLennan S, Elger BS. Nurses' attitudes towards enforced measures to increase influenza vaccination: A qualitative study. Influenza Other Respir Viruses. 2017;11(3):247-253. doi:10.1111/irv.12441
24	Luo Q, Gan L, Xiong Y, Li Q, Chen T, Tang X. Knowledge, attitudes and practices related to influenza and influenza vaccine among healthcare workers in Chongqing, China-a cross-sectional study. Hum Vaccin Immunother. 2021;17(12):5500-5508. doi:10.1080/21645515.2021.2007013
25	Alsuhaibani M. Barriers and beliefs among health-care workers regarding seasonal influenza vaccine in Al-Qassim region, Saudi Arabia. Hum Vaccin Immunother. 2020;16(2):313-320. doi:10.1080/21645515.2019.1656020
26	Gafner M, Korlander H, Zimlichman R, Ziv-Baran T, Zimlichman E. Influenza Vaccination Rate and Factors Associated With Compliance Among Health Care Employees in Large and Medium Acute Care Hospitals. Am J Med Qual. 2021;36(2):115-121. doi:10.1177/1062860620929423
27	Lim DW, Ho HJ, Lee LT, Chow A, Kyaw WM. Determinants of change in intention to receive influenza vaccination among health-care workers in Singapore. Hum Vaccin Immunother. 2020;16(5):1118-1124. doi:10.1080/21645515.2019.1688037
28	Çiftci F, Şen E, Demir N, Çiftci O, Erol S, Kayacan O. Beliefs, attitudes, and activities of healthcare personnel about influenza and pneumococcal vaccines. Hum Vaccin Immunother. 2018;14(1):111-117. doi:10.1080/21645515.2017.1387703
29	Akan H, Yavuz E, Yayla ME, et al. Factors affecting uptake of influenza vaccination among family physicians. Vaccine. 2016;34(14):1712-1718. doi:10.1016/j.vaccine.2016.01.057
30	Maridor M, Ruch S, Bangerter A, Emery V. Skepticism toward Emerging Infectious Diseases and Influenza Vaccination Intentions in Nurses. J Health Commun. 2017;22(5):386-394. doi:10.1080/10810730.2017.1296509
31	Zielonka TM, Szymańczak M, Jakubiak J, Nitsch-Osuch A, Życińska K. Influenza Vaccination Coverage Rate for Medical Staff: Influence of Hospital-Based Vaccination Campaign. Adv Exp Med Biol. 2016;885:31-38. doi:10.1007/5584_2015_197
32	Quinn G. Nurses' experiences of the seasonal influenza vaccine in residential care. Br J Nurs. 2014;23(17):942-948. doi:10.12968/bjon.2014.23.17.942
33	Pavlič DR, Maksuti A, Podnar B, Kokalj Kokot M. Reasons for the low influenza vaccination rate among nurses in Slovenia. Prim Health Care Res Dev. 2020;21:e38. Published 2020 Sep 30. doi:10.1017/S1463423620000419
34	Awali RA, Samuel PS, Marwaha B, et al. Understanding health care personnel's attitudes toward mandatory influenza vaccination. Am J Infect Control. 2014;42(6):649-652. doi:10.1016/j.ajic.2014.02.025
35	Assaf AM, Hammad EA, Haddadin RN. Influenza Vaccination Coverage Rates, Knowledge, Attitudes, and Beliefs in Jordan: A Comprehensive Study. Viral Immunol. 2016;29(9):516-525. doi:10.1089/vim.2015.0135
36	Khan TM, Khan AU, Ali I, Wu DB. Knowledge, attitude and awareness among healthcare professionals about influenza vaccination in Peshawar, Pakistan. Vaccine. 2016;34(11):1393-1398. doi:10.1016/j.vaccine.2016.01.045
37	Zhang J, While AE, Norman IJ. Nurses' vaccination against pandemic H1N1 influenza and their knowledge and other factors. Vaccine. 2012;30(32):4813-4819. doi:10.1016/j.vaccine.2012.05.012
38	Hudu SA, Harmal NS, Malina O, Sekawi Z. Influenza vaccination among Malaysian healthcare workers: a survey of coverage and attitudes. Med J Malaysia. 2016;71(5):231-237.

39	Takayanagi IJ, Cardoso MR, Costa SF, Araya ME, Machado CM. Attitudes of health care workers to influenza vaccination: why are they not vaccinated?. Am J Infect Control. 2007;35(1):56-61. doi:10.1016/j.ajic.2006.06.002
40	Asma S, Akan H, Uysal Y, et al. Factors effecting influenza vaccination uptake among health care workers: a multi-center cross-sectional study. BMC Infect Dis. 2016;16:192. Published 2016 May 4. doi:10.1186/s12879-016-1528-9
41	Keske Ş, Mutters NT, Tsioutis C, Ergönül Ö; EUCIC influenza vaccination survey team. Influenza vaccination among infection control teams: A EUCIC survey prior to COVID-19 pandemic. Vaccine. 2020;38(52):8357-8361. doi:10.1016/j.vaccine.2020.11.003
42	Shahar I, Mendelson G, Ben Natan M. Intention to receive the seasonal influenza vaccine among nurses working in a long-term care facility. Int J Nurs Pract. 2017;23(2):10.1111/ijn.12512. doi:10.1111/ijn.12512
43	Levi M, Bonanni P, Biffino M, et al. Influenza vaccination 2014-2015: Results of a survey conducted among general practitioners in Italy. Hum Vaccin Immunother. 2018;14(6):1342-1350. doi:10.1080/21645515.2018.1430543
44	Godin G, Vézina-Im LA, Naccache H. Determinants of influenza vaccination among healthcare workers. Infect Control Hosp Epidemiol. 2010;31(7):689-693. doi:10.1086/653614
45	Millner VS, Eichold BH 2nd, Franks RD, Johnson GD. Influenza vaccination acceptance and refusal rates among health care personnel. South Med J. 2010;103(10):993-998. doi:10.1097/SMJ.0b013e3181eda3d5
46	Andayi F, Emukule GO, Osoro E, et al. Knowledge and attitude of Kenyan healthcare workers towards pandemic influenza disease and vaccination: 9 years after the last influenza pandemic. Vaccine. 2021;39(29):3991-3996. doi:10.1016/j.vaccine.2021.05.057
47	Rebmann T, Wright KS, Anthony J, Knaup RC, Peters EB. Seasonal influenza vaccine compliance among hospital-based and nonhospital-based healthcare workers. Infect Control Hosp Epidemiol. 2012;33(3):243-249. doi:10.1086/664057
48	Bazán M, Villacorta E, Barbagelatta G, et al. Health workers' attitudes, perceptions and knowledge of influenza immunization in Lima, Peru: A mixed methods study. Vaccine. 2017;35(22):2930-2936. doi:10.1016/j.vaccine.2017.04.021
49	Benin AL, Lockwood G, Creatore T, Donovan D, Predmore M, MacArthur S. Improving Mandatory Vaccination Against Influenza: Minimizing Anxiety of Employees to Maximize Health of Patients. Am J Med Qual. 2018;33(4):372-382. doi:10.1177/1062860617748738
50	Hubble MW, Zontek TL, Richards ME. Predictors of influenza vaccination among emergency medical services personnel. Prehosp Emerg Care. 2011;15(2):175-183. doi:10.3109/10903127.2010.541982
51	Alshammari TM, AlFehaid LS, AlFraih JK, Aljadhey HS. Health care professionals' awareness of, knowledge about and attitude to influenza vaccination. Vaccine. 2014;32(45):5957-5961. doi:10.1016/j.vaccine.2014.08.061
52	Seale H, Wang Q, Yang P, et al. Influenza vaccination amongst hospital health care workers in Beijing. Occup Med (Lond). 2010;60(5):335-339. doi:10.1093/occmed/kqq037
53	Muhammad HS, Gueret P, Hayes B. Attitudes of Hospital Healthcare Workers towards Influenza Vaccination in a Tertiary Hospital Setting. Ir Med J. 2015;108(6):185-187.
54	Wicker S, Rabenau HF, Doerr HW, Allwinn R. Influenza vaccination compliance among health care workers in a German university hospital. Infection. 2009;37(3):197-202. doi:10.1007/s15010-008-8200-2

55	Oguz MM. Improving influenza vaccination uptake among healthcare workers by on-site influenza vaccination campaign in a tertiary children hospital. Hum Vaccin Immunother. 2019;15(5):1060-1065. doi:10.1080/21645515.2019.1575164
56	Rehmani R, Memon JI. Knowledge, attitudes and beliefs regarding influenza vaccination among healthcare workers in a Saudi hospital. Vaccine. 2010;28(26):4283-4287. doi:10.1016/j.vaccine.2010.04.031
57	Savas E, Tanriverdi D. Knowledge, attitudes and anxiety towards influenza A/H1N1 vaccination of healthcare workers in Turkey. BMC Infect Dis. 2010;10:281. Published 2010 Sep 23. doi:10.1186/1471-2334-10-281
58	Kent JN, Lea CS, Fang X, Novick LF, Morgan J. Seasonal influenza vaccination coverage among local health department personnel in North Carolina, 2007-2008. Am J Prev Med. 2010;39(1):74-77. doi:10.1016/j.amepre.2010.03.007
59	Jaiyeoba O, Villers M, Soper DE, Korte J, Salgado CD. Association between health care workers' knowledge of influenza vaccine and vaccine uptake. Am J Infect Control. 2014;42(1):69-70. doi:10.1016/j.ajic.2013.06.020
60	Cheung EKH, Lee S, Lee SS. Pattern of exposure to information and its impact on seasonal influenza vaccination uptake in nurses. J Hosp Infect. 2017;97(4):376-383. doi:10.1016/j.jhin.2017.08.005
61	Nowrouzi-Kia B, McGeer A. External cues to action and influenza vaccination among post-graduate trainee physicians in Toronto, Canada. Vaccine. 2014;32(30):3830-3834. doi:10.1016/j.vaccine.2014.04.067
62	Chor JS, Pada SK, Stephenson I, et al. Seasonal influenza vaccination predicts pandemic H1N1 vaccination uptake among healthcare workers in three countries. Vaccine. 2011;29(43):7364-7369. doi:10.1016/j.vaccine.2011.07.079
63	Raftopoulos V. Attitudes of nurses in Greece towards influenza vaccination. Nurs Stand. 2008;23(4):35-42. doi:10.7748/ns2008.10.23.4.35.c6675
64	Domínguez A, Godoy P, Castilla J, et al. Knowledge of and attitudes to influenza vaccination in healthy primary healthcare workers in Spain, 2011-2012. PLoS One. 2013;8(11):e81200. Published 2013 Nov 18. doi:10.1371/journal.pone.0081200
65	Mignot A, Wilhelm MC, Valette A, Gavard-Perret ML, Abord-De-Chatillon E, Epaulard O. Behavior of nurses and nurse aides toward influenza vaccine: the impact of the perception of occupational working conditions. Hum Vaccin Immunother. 2020;16(5):1125-1131. doi:10.1080/21645515.2019.1694328
66	Hidiroglu S, Ay P, Topuzoglu A, Kalafat C, Karavus M. Resistance to vaccination: the attitudes and practices of primary healthcare workers confronting the H1N1 pandemic. Vaccine. 2010;28(51):8120-8124. doi:10.1016/j.vaccine.2010.09.104
67	deSante JE, Caplan A, Shofer F, Behrman AJ. Physician attitudes towards influenza immunization and vaccine mandates. Vaccine. 2010;28(13):2517-2521. doi:10.1016/j.vaccine.2010.01.042
68	Abramson ZH, Avni O, Levi O, Miskin IN. Randomized trial of a program to increase staff influenza vaccination in primary care clinics. Ann Fam Med. 2010;8(4):293-298. doi:10.1370/afm.1132
69	Seale H, Leask J, MacIntyre CR. Attitudes amongst Australian hospital healthcare workers towards seasonal influenza and vaccination. Influenza Other Respir Viruses. 2010;4(1):41-46. doi:10.1111/j.1750-2659.2009.00112.x
70	Opstelten W, van Essen GA, Ballieux MJ, Goudswaard AN. Influenza immunization of Dutch general practitioners: vaccination rate and attitudes towards vaccination. Vaccine. 2008;26(47):5918-5921. doi:10.1016/j.vaccine.2008.08.049

71	Bali NK, Ashraf M, Ahmad F, et al. Knowledge, attitude, and practices about the seasonal influenza vaccination among healthcare workers in Srinagar, India. Influenza Other Respir Viruses. 2013;7(4):540-545. doi:10.1111/j.1750-2659.2012.00416.x
72	Cowan AE, Winston CA, Davis MM, Wortley PM, Clark SJ. Influenza vaccination status and influenza-related perspectives and practices among US physicians. Am J Infect Control. 2006;34(4):164-169. doi:10.1016/j.ajic.2005.09.007
73	Zimmerman RK, Nowalk MP, Lin CJ, et al. Factorial design for improving influenza vaccination among employees of a large health system. Infect Control Hosp Epidemiol. 2009;30(7):691-697. doi:10.1086/598343
74	Henriksen Hellyer JM, DeVries AS, Jenkins SM, et al. Attitudes toward and uptake of H1N1 vaccine among health care workers during the 2009 H1N1 pandemic. PLoS One. 2011;6(12):e29478. doi:10.1371/journal.pone.0029478
75	Norton SP, Scheifele DW, Bettinger JA, West RM. Influenza vaccination in paediatric nurses: cross-sectional study of coverage, refusal, and factors in acceptance. Vaccine. 2008;26(23):2942-2948. doi:10.1016/j.vaccine.2008.03.033
76	Barrière J, Vanjak D, Kriegel I, et al. Acceptance of the 2009 A(H1N1) influenza vaccine among hospital workers in two French cancer centers. Vaccine. 2010;28(43):7030-7034. doi:10.1016/j.vaccine.2010.08.021
77	Castilla J, Martínez-Baz I, Godoy P, et al. Trends in influenza vaccine coverage among primary healthcare workers in Spain, 2008-2011. Prev Med. 2013;57(3):206-211. doi:10.1016/j.ypmed.2013.05.021
78	Zhang J, While AE, Norman IJ. Seasonal influenza vaccination knowledge, risk perception, health beliefs and vaccination behaviours of nurses. Epidemiol Infect. 2012;140(9):1569-1577. doi:10.1017/S0950268811002214
79	Kopsidas I, Tsopela GC, Maroudi-Manta S, et al. Increasing healthcare workers' uptake of seasonal influenza vaccination in a tertiary-care pediatric hospital in Greece with a low-cost, tailor-made, multifaceted strategy. Vaccine. 2020;38(29):4609-4615. doi:10.1016/j.vaccine.2020.05.021
80	Weingarten S, Riedinger M, Bolton LB, Miles P, Ault M. Barriers to influenza vaccine acceptance. A survey of physicians and nurses. Am J Infect Control. 1989;17(4):202-207. doi:10.1016/0196-6553(89)90129-6
81	Marentette T, El-Masri MM. Predicting seasonal influenza vaccination among hospital-based nurses. Clin Nurs Res. 2011;20(4):422-438. doi:10.1177/1054773811409032
82	Goldstein AO, Kincade JE, Gamble G, Bearman RS. Policies and practices for improving influenza immunization rates among healthcare workers. Infect Control Hosp Epidemiol. 2004;25(11):908-911. doi:10.1086/502318
83	Ertugrul A, Sari E, Gulenc N, Ozmen S. Why are influenza vaccination coverage rates still too low among health care workers in a tertiary care children's hospital in Turkey?. J Public Health Policy. 2021;42(1):41-52. doi:10.1057/s41271-020-00250-1
84	Tapiainen T, Bär G, Schaad UB, Heininger U. Influenza vaccination among healthcare workers in a university children's hospital. Infect Control Hosp Epidemiol. 2005;26(11):855-858. doi:10.1086/502508
85	Canning HS, Phillips J, Allsup S. Health care worker beliefs about influenza vaccine and reasons for non-vaccination--a cross-sectional survey. J Clin Nurs. 2005;14(8):922-925. doi:10.1111/j.1365-2702.2005.01190.x
86	Toh MP, Kannan P, Chen Y, Chng FL, Tang WE. Healthcare workers and H1N1 vaccination: does having a chronic disease make a difference?. Vaccine. 2012;30(6):1064-1070. doi:10.1016/j.vaccine.2011.12.037

87	Toledo D, Soldevila N, Guayta-Escolies R, et al. Knowledge of and Attitudes to Influenza Vaccination among Community Pharmacists in Catalonia (Spain). 2013-2014 Season: A Cross Sectional Study. Int J Environ Res Public Health. 2017;14(7):756. Published 2017 Jul 11. doi:10.3390/ijerph14070756
88	Song JY, Park CW, Jeong HW, Cheong HJ, Kim WJ, Kim SR. Effect of a hospital campaign for influenza vaccination of healthcare workers. Infect Control Hosp Epidemiol. 2006;27(6):612-617. doi:10.1086/504503
89	Dhont PA, Albert A, Brenders P, et al. Acceptability of Intanza® 15 μg intradermal influenza vaccine in Belgium during the 2010-2011 influenza season. Adv Ther. 2012;29(6):562-577. doi:10.1007/s12325-012-0025-9
90	O'Reilly FW, Cran GW, Stevens AB. Factors affecting influenza vaccine uptake among health care workers. Occup Med (Lond). 2005;55(6):474-479. doi:10.1093/occmed/kqi099
91	Mak KK, Yiu YF, Ko KL, et al. Attitudes and perceptions of influenza vaccination among Hong Kong doctors and medical students before the 2009 pandemic. Eur J Public Health. 2013;23(2):257-262. doi:10.1093/eurpub/cks014
92	To KW, Lee S, Chan TO, Lee SS. Exploring determinants of acceptance of the pandemic influenza A (H1N1) 2009 vaccination in nurses. Am J Infect Control. 2010;38(8):623-630. doi:10.1016/j.ajic.2010.05.015
93	Prematunge C, Corace K, McCarthy A, et al. Qualitative motivators and barriers to pandemic vs. seasonal influenza vaccination among healthcare workers: a content analysis. Vaccine. 2014;32(52):7128-7134. doi:10.1016/j.vaccine.2014.10.023
94	Kaufman J, Davis J, Krause V. Influenza immunisation of doctors at an Australian tertiary hospital: immunisation rate and factors contributing to uptake. Commun Dis Intell Q Rep. 2008;32(4):443-448.
95	Sartor C, Tissot-Dupont H, Zandotti C, Martin F, Roques P, Drancourt M. Use of a mobile cart influenza program for vaccination of hospital employees. Infect Control Hosp Epidemiol. 2004;25(11):918-922. doi:10.1086/502320
96	Boey L, Roelants M, Vandermeulen C. Increased vaccine uptake and less perceived barriers toward vaccination in long-term care facilities that use multi-intervention manual for influenza campaigns. Hum Vaccin Immunother. 2021;17(3):673-680. doi:10.1080/21645515.2020.1788327
97	Rueckmann E, Shah MN, Humiston SG. Influenza vaccination among emergency medical services and emergency department personnel. Prehosp Emerg Care. 2009;13(1):1-5. doi:10.1080/10903120802471949
98	Durando P, Alicino C, Dini G, et al. Determinants of adherence to seasonal influenza vaccination among healthcare workers from an Italian region: results from a cross-sectional study. BMJ Open. 2016;6(5):e010779. Published 2016 May 17. doi:10.1136/bmjopen-2015-010779
99	Edge R, Keegan T, Isba R, Diggle P. Observational study to assess the effects of social networks on the seasonal influenza vaccine uptake by early career doctors. BMJ Open. 2019;9(8):e026997. Published 2019 Aug 30. doi:10.1136/bmjopen-2018-026997
100	Ruiz AD, Frei CR, Barner JC, et al. Influenza vaccination rates among pharmacists. J Am Pharm Assoc (2003). 2010;50(4):517-522. doi:10.1331/JAPhA.2010.09146
101	Doratotaj S, Macknin ML, Worley S. A novel approach to improve influenza vaccination rates among health care professionals: a prospective randomized controlled trial. Am J Infect Control. 2008;36(4):301-303. doi:10.1016/j.ajic.2007.10.019

102	Boey L, Bral C, Roelants M, et al. Attitudes, believes, determinants and organisational barriers behind the low seasonal influenza vaccination uptake in healthcare workers - A cross-sectional survey. Vaccine. 2018;36(23):3351-3358. doi:10.1016/j.vaccine.2018.04.044
103	Pavia M, Foresta MR, Carbone V, Angelillo IF. Influenza and pneumococcal immunization in the elderly: knowledge, attitudes, and practices among general practitioners in Italy. Public Health. 2003;117(3):202-207. doi:10.1016/s0033-3506(03)00066-0
104	Tanguy M, Boyeau C, Pean S, Marijon E, Delhumeau A, Fanello S. Acceptance of seasonal and pandemic a (H1N1) 2009 influenza vaccination by healthcare workers in a french teaching hospital. Vaccine. 2011;29(25):4190-4194. doi:10.1016/j.vaccine.2011.03.107
105	Kung YM. A quality improvement project to increase influenza vaccination in healthcare personnel at a university health center. J Am Assoc Nurse Pract. 2014;26(3):148-154. doi:10.1002/2327-6924.12060
106	Piccirillo B, Gaeta T. Survey on use of and attitudes toward influenza vaccination among emergency department staff in a New York metropolitan hospital. Infect Control Hosp Epidemiol. 2006;27(6):618-622. doi:10.1086/504448
107	Willis BC, Wortley P. Nurses' attitudes and beliefs about influenza and the influenza vaccine: a summary of focus groups in Alabama and Michigan. Am J Infect Control. 2007;35(1):20-24. doi:10.1016/j.ajic.2006.07.009
108	Desiante F, Caputi G, Cipriani R, et al. Assessment of coverage and analysis of the determinants of adherence to influenza vaccination in the general practitioners of Taranto. Ann Ig. 2017;29(4):256-263. doi:10.7416/ai.2017.2157
109	Ribner BS, Hall C, Steinberg JP, et al. Use of a mandatory declination form in a program for influenza vaccination of healthcare workers. Infect Control Hosp Epidemiol. 2008;29(4):302-308. doi:10.1086/529586
110	Rabensteiner A, Buja A, Regele D, Fischer M, Baldo V. Healthcare worker's attitude to seasonal influenza vaccination in the South Tyrolean province of Italy: barriers and facilitators. Vaccine. 2018;36(4):535-544. doi:10.1016/j.vaccine.2017.12.007
111	Beguin C, Boland B, Ninane J. Health care workers: vectors of influenza virus? Low vaccination rate among hospital health care workers. Am J Med Qual. 1998;13(4):223-227. doi:10.1177/106286069801300408
112	Arda B, Durusoy R, Yamazhan T, et al. Did the pandemic have an impact on influenza vaccination attitude? A survey among health care workers. BMC Infect Dis. 2011;11:87. Published 2011 Apr 7. doi:10.1186/1471-2334-11-87
113	Heinrich-Morrison K, McLellan S, McGinnes U, et al. An effective strategy for influenza vaccination of healthcare workers in Australia: experience at a large health service without a mandatory policy. BMC Infect Dis. 2015;15:42. Published 2015 Feb 6. doi:10.1186/s12879-015-0765-7
114	Godinot LD, Sicsic J, Lachatre M, et al. Quantifying preferences around vaccination against frequent, mild disease with risk for vulnerable persons: A discrete choice experiment among French hospital health care workers. Vaccine. 2021;39(5):805-814. doi:10.1016/j.vaccine.2020.12.057
115	Lehmann BA, Ruiter RA, van Dam D, Wicker S, Kok G. Sociocognitive predictors of the intention of healthcare workers to receive the influenza vaccine in Belgian, Dutch and German hospital settings. J Hosp Infect. 2015;89(3):202-209. doi:10.1016/j.jhin.2014.11.009
116	Pettke A, Jocham S, Wiener A, et al. Vaccination against influenza at a European pediatric cancer center: immunization rates and attitudes among staff, patients, and their families. Support Care Cancer. 2017;25(12):3815-3822. doi:10.1007/s00520-017-3813-6

117	Kaboli F, Astrakianakis G, Li G, Guzman J, Donovan T, Naus M. Influenza vaccination and intention to receive the pandemic H1N1 influenza vaccine among healthcare workers of British Columbia, Canada: a cross-sectional study [published correction appears in Infect Control Hosp Epidemiol. 2010 Dec;31(12):1316]. Infect Control Hosp Epidemiol. 2010;31(10):1017-1024. doi:10.1086/655465
118	Ali Imtiaz M, Budnick LD, Berman AR. Influenza immunization among resident physicians in an urban teaching hospital. Am J Infect Control. 2016;44(4):491-493. doi:10.1016/j.ajic.2015.10.029
119	Coulibaly D, Nzussouo NT, Kadjo HA, et al. Pandemic Influenza A(H1N1) in Cote d'Ivoire: health-care providers' knowledge of influenza and attitudes towards vaccination. J Infect Dev Ctries. 2013;7(7):499-506. Published 2013 Jul 15. doi:10.3855/jidc.2771
120	Cihan FG, Durmaz FG, Odabas D, Baydemir C, Fatma K. Attitudes toward and factors affecting influenza vaccination among physicians and nurses of a tertiary-care hospital in the Central Anatolia region of Turkey. Postgrad Med. 2012;124(6):117-123. doi:10.3810/pgm.2012.11.2602
121	Alsaleem MA. Acceptance of H1N1 vaccine among healthcare workers at primary healthcare centres in Abha, KSA. J Egypt Public Health Assoc. 2013;88(1):32-39. doi:10.1097/01.EPX.0000426292.89751.ca
122	Brunton C, Weir R, Jennings L; National Influenza and Pneumococcal Immunisation Attitudes Study (NIPIAS) Group. Knowledge and attitudes about influenza vaccination amongst general practitioners, practice nurses, and people aged 65 and over. N Z Med J. 2004;118(1214):U1434. Published 2004 May 6.
123	Fernández-Villa T, Molina AJ, Torner N, et al. Factors associated with acceptance of pandemic flu vaccine by healthcare professionals in Spain, 2009-2010. Res Nurs Health. 2017;40(5):435-443. doi:10.1002/nur.21815
124	Steiner M, Vermeulen LC, Mullahy J, Hayney MS. Factors influencing decisions regarding influenza vaccination and treatment: a survey of healthcare workers. Infect Control Hosp Epidemiol. 2002;23(10):625-627. doi:10.1086/501984
125	Wong MCS, Nelson EAS, Leung C, et al. Ad Hoc Influenza Vaccination During Years of Significant Antigenic Drift in a Tropical City With 2 Seasonal Peaks: A Cross-Sectional Survey Among Health Care Practitioners. Medicine (Baltimore). 2016;95(19):e3359. doi:10.1097/MD.0000000000003359
126	Fatiregun AA, Adeyemo AA, Olowookere SA. Willingness to receive pandemic influenza A (H1N1) vaccine among doctors and nurses in public health facilities in Ibadan, Nigeria. Vaccine. 2012;30(13):2315-2319. doi:10.1016/j.vaccine.2012.01.060
127	Shroufi A, Copping J, Musonda P, et al. Influenza vaccine uptake among staff in care homes in Nottinghamshire: a random cluster sample survey. Public Health. 2009;123(10):645-649. doi:10.1016/j.puhe.2009.09.014
128	Bonfiglioli R, Vignoli M, Guglielmi D, Depolo M, Violante FS. Getting vaccinated or not getting vaccinated? Different reasons for getting vaccinated against seasonal or pandemic influenza. BMC Public Health. 2013;13:1221. Published 2013 Dec 21. doi:10.1186/1471-2458-13-1221
129	Kimura AC, Nguyen CN, Higa JI, Hurwitz EL, Vugia DJ. The effectiveness of vaccine day and educational interventions on influenza vaccine coverage among health care workers at long-term care facilities. Am J Public Health. 2007;97(4):684-690. doi:10.2105/AJPH.2005.082073

130	Schwarzinger M, Verger P, Guerville MA, et al. Positive attitudes of French general practitioners towards A/H1N1 influenza-pandemic vaccination: a missed opportunity to increase vaccination uptakes in the general public?. Vaccine. 2010;28(15):2743-2748. doi:10.1016/j.vaccine.2010.01.027
131	Amodio E, Tramuto F, Maringhini G, et al. Are medical residents a "core group" for future improvement of influenza vaccination coverage in health-care workers? A study among medical residents at the University Hospital of Palermo (Sicily). Vaccine. 2011;29(45):8113-8117. doi:10.1016/j.vaccine.2011.08.033
132	Kraut A, Graff L, McLean D. Behavioral change with influenza vaccination: factors influencing increased uptake of the pandemic H1N1 versus seasonal influenza vaccine in health care personnel. Vaccine. 2011;29(46):8357-8363. doi:10.1016/j.vaccine.2011.08.084
133	Shah SI, Caprio M. Availability of trivalent inactivated influenza vaccine to parents of neonatal intensive care unit patients and its effect on the healthcare worker vaccination rate. Infect Control Hosp Epidemiol. 2008;29(4):309-313. doi:10.1086/527515
134	Massin S, Ventelou B, Nebout A, Verger P, Pulcini C. Cross-sectional survey: risk-averse French general practitioners are more favorable toward influenza vaccination. Vaccine. 2015;33(5):610-614. doi:10.1016/j.vaccine.2014.12.038
135	Marshall RJ, Tetu-Mouradjian LM, Fulton JP. Increasing annual influenza vaccinations among healthcare workers in Rhode Island: a social marketing approach. Med Health R I. 2010;93(9):271-278.
136	Brandt C, Rabenau HF, Bornmann S, Gottschalk R, Wicker S. The impact of the 2009 influenza A(H1N1) pandemic on attitudes of healthcare workers toward seasonal influenza vaccination 2010/11. Euro Surveill. 2011;16(17):19854. Published 2011 Apr 28.
137	Seale H, Kaur R, Wang Q, et al. Acceptance of a vaccine against pandemic influenza A (H1N1) virus amongst healthcare workers in Beijing, China. Vaccine. 2011;29(8):1605-1610. doi:10.1016/j.vaccine.2010.12.077
138	Madewell ZJ, Chacón-Fuentes R, Jara J, et al. Knowledge, attitudes, and practices of seasonal influenza vaccination in healthcare workers, Honduras. PLoS One. 2021;16(2):e0246379. Published 2021 Feb 4. doi:10.1371/journal.pone.0246379
139	Torun SD, Torun F. Vaccination against pandemic influenza A/H1N1 among healthcare workers and reasons for refusing vaccination in Istanbul in last pandemic alert phase. Vaccine. 2010;28(35):5703-5710. doi:10.1016/j.vaccine.2010.06.049
140	Feemster KA, Prasad P, Smith MJ, et al. Employee designation and health care worker support of an influenza vaccine mandate at a large pediatric tertiary care hospital. Vaccine. 2011;29(9):1762-1769. doi:10.1016/j.vaccine.2010.12.115
141	Fernandez WG, Oyama L, Mitchell P, et al. Attitudes and practices regarding influenza vaccination among emergency department personnel. J Emerg Med. 2009;36(2):201-206. doi:10.1016/j.jemermed.2007.07.070
142	Ballada D, Biasio LR, Cascio G, et al. Attitudes and behavior of health care personnel regarding influenza vaccination. Eur J Epidemiol. 1994;10(1):63-68. doi:10.1007/BF01717454
143	Yang KS, Fong YT, Koh D, Lim MK. High coverage of influenza vaccination among healthcare workers can be achieved during heightened awareness of impending threat. Ann Acad Med Singap. 2007;36(6):384-387.
144	Heininger U, Bächler M, Schaad UB. Attitudes of pediatricians regarding influenza self-immunization: a survey in a Swiss university children's hospital. Pediatr Infect Dis J. 2003;22(5):391-394. doi:10.1097/01.inf.0000066901.59298.a8

145	Esteves-Jaramillo A, Omer SB, Gonzalez-Diaz E, et al. Acceptance of a vaccine against novel influenza A (H1N1) virus among health care workers in two major cities in Mexico. Arch Med Res. 2009;40(8):705-711. doi:10.1016/j.arcmed.2010.01.004
146	Corace K, Prematunge C, McCarthy A, et al. Predicting influenza vaccination uptake among health care workers: what are the key motivators?. Am J Infect Control. 2013;41(8):679-684. doi:10.1016/j.ajic.2013.01.014
147	Madewell Z, Chacón-Fuentes R, Badilla-Vargas X, et al. Knowledge, attitudes, and practices for the use of seasonal influenza vaccination, healthcare workers, Costa Rica. J Infect Dev Ctries. 2021;15(7):1004-1013. Published 2021 Jul 31. doi:10.3855/jidc.14381
148	Ofstead CL, Tucker SJ, Beebe TJ, Poland GA. Influenza vaccination among registered nurses: information receipt, knowledge, and decision-making at an institution with a multifaceted educational program. Infect Control Hosp Epidemiol. 2008;29(2):99-106. doi:10.1086/526431
149	Looijmans-van den Akker I, van Delden JJ, Verheij TJ, et al. Which determinants should be targeted to increase influenza vaccination uptake among health care workers in nursing homes?. Vaccine. 2009;27(34):4724-4730. doi:10.1016/j.vaccine.2009.05.013
150	Bodelet C, Bodelet J, Landelle C, Gauchet A. Seasonal flu vaccination, a matter of emotion? An experimental study on role of compassion, socioeconomic status and perceived threat among healthcare workers. Psychol Health. 2021;36(12):1461-1479. doi:10.1080/08870446.2020.1856843
151	Yi H, Yang Y, Zhang L, et al. Improved influenza vaccination coverage among health-care workers: evidence from a web-based survey in China, 2019/2020 season. Hum Vaccin Immunother. 2021;17(7):2185-2189. doi:10.1080/21645515.2020.1859317
152	Ng TWY, Cowling BJ, So HC, Ip DKM, Liao Q. Testing an integrative theory of health behavioural change for predicting seasonal influenza vaccination uptake among healthcare workers. Vaccine. 2020;38(3):690-698. doi:10.1016/j.vaccine.2019.10.041
153	Surtees TC, Teh BW, Slavin MA, Worth LJ. Factors contributing to declination of annual influenza vaccination by healthcare workers caring for cancer patients: An Australian experience. Vaccine. 2018;36(14):1804-1807. doi:10.1016/j.vaccine.2018.02.098
154	Gianfredi V, Nucci D, Salvatori T, et al. "PErCEIVE in Umbria": evaluation of anti-influenza vaccination's perception among Umbrian pharmacists. J Prev Med Hyg. 2018;59(1):E14-E19. Published 2018 Mar 30. doi:10.15167/2421-4248/jpmh2018.59.1.806
155	Rebmann T, Wright KS, Anthony J, Knaup RC, Peters EB. Seasonal and H1N1 influenza vaccine compliance and intent to be vaccinated among emergency medical services personnel. Am J Infect Control. 2012;40(7):632-636. doi:10.1016/j.ajic.2011.12.016
156	Winston L, Wagner S, Chan S. Healthcare workers under a mandated H1N1 vaccination policy with employment termination penalty: a survey to assess employee perception. Vaccine. 2014;32(37):4786-4790. doi:10.1016/j.vaccine.2014.06.001
157	Real K, Kim S, Conigliaro J. Using a validated health promotion tool to improve patient safety and increase health care personnel influenza vaccination rates. Am J Infect Control. 2013;41(8):691-696. doi:10.1016/j.ajic.2012.09.027
158	Tagajdid MR, El Annaz H, Belefquih B, et al. Factors influencing uptake of influenza vaccine amongst healthcare workers in a regional center after the A(H1N1) 2009 pandemic: lessons for improving vaccination rates. Int J Risk Saf Med. 2011;23(4):249-254. doi:10.3233/JRS-2011-0544
159	Rhudy LM, Tucker SJ, Ofstead CL, Poland GA. Personal choice or evidence-based nursing intervention: nurses' decision-making about influenza vaccination. Worldviews Evid Based Nurs. 2010;7(2):111-120. doi:10.1111/j.1741-6787.2010.00190.x

160	Tam DK, Lee SS, Lee S. Impact of severe acute respiratory syndrome and the perceived avian influenza epidemic on the increased rate of influenza vaccination among nurses in Hong Kong. Infect Control Hosp Epidemiol. 2008;29(3):256-261. doi:10.1086/527507
161	Lehmann BA, Ruiter RAC, Chapman G, Kok G. The intention to get vaccinated against influenza and actual vaccination uptake of Dutch healthcare personnel. Vaccine. 2014;32(51):6986-6991. doi:10.1016/j.vaccine.2014.10.034
162	Ehrenstein BP, Hanses F, Blaas S, Mandraka F, Audebert F, Salzberger B. Perceived risks of adverse effects and influenza vaccination: a survey of hospital employees. Eur J Public Health. 2010;20(5):495-499. doi:10.1093/eurpub/ckp227
163	Choucair K, El Sawda J, Assaad S, et al. Knowledge, Perception, Attitudes and Behavior on Influenza Immunization and the Determinants of Vaccination. J Epidemiol Glob Health. 2021;11(1):34-41. doi:10.2991/jegh.k.200906.001
164	Tao XG, Giampino J, Dooley DA, Humphrey FE, Baron DM, Bernacki EJ. Description of an influenza vaccination campaign and use of a randomized survey to determine participation rates. Infect Control Hosp Epidemiol. 2010;31(2):151-157. doi:10.1086/649798
165	Pinto L, Falsaperla R, Villani A, et al. Influenza vaccination: opinions of health care professionals working in pediatric emergency departments. Ital J Pediatr. 2019;45(1):47. Published 2019 Apr 11. doi:10.1186/s13052-019-0638-6
166	Little KE, Goodridge S, Lewis H, et al. Occupational vaccination of health care workers: uptake, attitudes and potential solutions. Public Health. 2015;129(6):755-762. doi:10.1016/j.puhe.2015.02.031
167	Hill JN, Smith BM, Evans CT, Anaya H, Goldstein B, LaVela SL. Implementing a declination form programme to improve influenza vaccine uptake by staff in Department of Veterans Affairs spinal cord injury centres: a pilot study. J Hosp Infect. 2015;91(2):158-165. doi:10.1016/j.jhin.2015.05.015
168	Hagemeister MH, Stock NK, Ludwig T, Heuschmann P, Vogel U. Self-reported influenza vaccination rates and attitudes towards vaccination among health care workers: results of a survey in a German university hospital. Public Health. 2018;154:102-109. doi:10.1016/j.puhe.2017.10.027
169	Atamna Z, Chazan B, Nitzan O, et al. Seasonal Influenza Vaccination Effectiveness and Compliance among Hospital Health Care Workers. Isr Med Assoc J. 2016;18(1):5-9.
170	Valour F, Bénet T, Chidiac C; Study group. Pandemic A(H1N1)2009 influenza vaccination in Lyon University Hospitals, France: perception and attitudes of hospital workers. Vaccine. 2013;31(4):592-595. doi:10.1016/j.vaccine.2012.11.070
171	Elias C, Fournier A, Vasiliu A, et al. Seasonal influenza vaccination coverage and its determinants among nursing homes personnel in western France. BMC Public Health. 2017;17(1):634. Published 2017 Jul 7. doi:10.1186/s12889-017-4556-5
172	McEwen M, Farren E. Actions and beliefs related to hepatitis B and influenza immunization among registered nurses in Texas. Public Health Nurs. 2005;22(3):230-239. doi:10.1111/j.0737-1209.2005.220306.x
173	Hajiabdolbaghi M, Havastin NG, Afhami S, et al. Influenza vaccination coverage and obstacles in healthcare workers (HCWs) and the follow up of side effects: a multicenter investigation in Iran. J Prev Med Hyg. 2021;62(2):E377-E381. Published 2021 Jul 30. doi:10.15167/2421-4248/jpmh2021.62.2.1827
174	Banach DB, Zhang C, Factor SH, Calfee DP. Support for mandatory health care worker influenza vaccination among allied health professionals, technical staff, and medical students. Am J Infect Control. 2013;41(4):354-356. doi:10.1016/j.ajic.2012.05.019

175	Halliday L, Thomson JA, Roberts L, Bowen S, Mead C. Influenza vaccination of staff in aged care facilities in the ACT: how can we improve the uptake of influenza vaccine?. Aust N Z J Public Health. 2003;27(1):70-75. doi:10.1111/j.1467-842x.2003.tb00383.x
176	Gur-Arie R, Katz MA, Hirsch A, et al. "You Have to Die Not to Come to Work": A Mixed Methods Study of Attitudes and Behaviors regarding Presenteeism, Absenteeism and Influenza Vaccination among Healthcare Personnel with Respiratory Illness in Israel, 2016-2019. Vaccine. 2021;39(17):2366-2374. doi:10.1016/j.vaccine.2021.03.057
177	Eisenfeld L, Perl L, Burke G, et al. Lack of compliance with influenza immunization for caretakers of neonatal intensive care unit patients. Am J Infect Control. 1994;22(5):307-311. doi:10.1016/0196-6553(94)90018-3
178	Hakim H, Gaur AH, McCullers JA. Motivating factors for high rates of influenza vaccination among healthcare workers. Vaccine. 2011;29(35):5963-5969. doi:10.1016/j.vaccine.2011.06.041
179	Heimberger T, Chang HG, Shaikh M, Crotty L, Morse D, Birkhead G. Knowledge and attitudes of healthcare workers about influenza: why are they not getting vaccinated?. Infect Control Hosp Epidemiol. 1995;16(7):412-415. doi:10.1086/647139
180	Chor JS, Ngai KL, Goggins WB, et al. Willingness of Hong Kong healthcare workers to accept pre-pandemic influenza vaccination at different WHO alert levels: two questionnaire surveys. BMJ. 2009;339:b3391. Published 2009 Aug 25. doi:10.1136/bmj.b3391
181	Albanesi B, Clari M, Gonella S, et al. The impact of COVID-19 on hospital-based workers influenza vaccination uptake: A two-year retrospective cohort study [published correction appears in J Occup Health. 2023 Jan;65(1):e12394]. J Occup Health. 2022;64(1):e12376. doi:10.1002/1348-9585.12376
182	Lim DW, Lee LT, Kyaw WM, Chow A. Psychosocial determinants of influenza vaccination intention: A cross-sectional study on inpatient nurses in Singapore. Am J Infect Control. 2017;45(10):e115-e117. doi:10.1016/j.ajic.2017.03.017
183	Naz H, Cevik F, Aykin N. Influenza vaccination in healthcare workers. J Infect Dev Ctries. 2009;3(1):50-54. Published 2009 Feb 28. doi:10.3855/jidc.105
184	Blasi F, Palange P, Rohde G, Severin T, Cornaglia G, Finch R. Healthcare workers and influenza vaccination: an ERS-ESCMID Web-based survey. Clin Microbiol Infect. 2011;17(8):1223-1225. doi:10.1111/j.1469-0691.2011.03501.x
185	Rubin GJ, Potts HW, Michie S. Likely uptake of swine and seasonal flu vaccines among healthcare workers. A cross-sectional analysis of UK telephone survey data. Vaccine. 2011;29(13):2421-2428. doi:10.1016/j.vaccine.2011.01.035
186	Rashid ZZ, Jasme H, Liang HJ, et al. INFLUENZA VACCINATION UPTAKE AMONG HEALTHCARE WORKERS AT A MALAYSIAN TEACHING HOSPITAL. Southeast Asian J Trop Med Public Health. 2015;46(2):215-225.
187	Song Y, Zhang T, Chen L, et al. Increasing seasonal influenza vaccination among high risk groups in China: Do community healthcare workers have a role to play?. Vaccine. 2017;35(33):4060-4063. doi:10.1016/j.vaccine.2017.06.054
188	Dorribo V, Lazor-Blanchet C, Hugli O, Zanetti G. Health care workers' influenza vaccination: motivations and mandatory mask policy. Occup Med (Lond). 2015;65(9):739-745. doi:10.1093/occmed/kqv116
189	Hirsch P, Hodgson M, Davey V. Seasonal influenza vaccination of healthcare employees: results of a 4-year campaign. Infect Control Hosp Epidemiol. 2011;32(5):444-448. doi:10.1086/659764

190	Wang K, Wong ELY, Ho KF, et al. Intention of nurses to accept coronavirus disease 2019 vaccination and change of intention to accept seasonal influenza vaccination during the coronavirus disease 2019 pandemic: A cross-sectional survey. Vaccine. 2020;38(45):7049-7056. doi:10.1016/j.vaccine.2020.09.021
191	Costantino C, Restivo V, Gaglio V, et al. Effectiveness of an educational intervention on seasonal influenza vaccination campaign adherence among healthcare workers of the Palermo University Hospital, Italy. Ann Ig. 2019;31(1):35-44. doi:10.7416/ai.2019.2256
192	Kyaw WM, Chow A, Hein AA, Lee LT, Leo YS, Ho HJ. Factors influencing seasonal influenza vaccination uptake among health care workers in an adult tertiary care hospital in Singapore: A cross-sectional survey. Am J Infect Control. 2019;47(2):133-138. doi:10.1016/j.ajic.2018.08.011
193	Attinsounon CA, Cordonnier C, Pulcini C, Di-Patrizio P, Thilly N, May T. Factors associated with influenza vaccination of general medicine interns in Nancy, France, in 2017. Eur J Clin Microbiol Infect Dis. 2019;38(12):2267-2273. doi:10.1007/s10096-019-03669-x
194	Maltezou HC, Dedoukou X, Patrinos S, et al. Determinants of intention to get vaccinated against novel (pandemic) influenza A H1N1 among health-care workers in a nationwide survey. J Infect. 2010;61(3):252-258. doi:10.1016/j.jinf.2010.06.004
195	Naleway AL, Henkle EM, Ball S, et al. Barriers and facilitators to influenza vaccination and vaccine coverage in a cohort of health care personnel. Am J Infect Control. 2014;42(4):371-375. doi:10.1016/j.ajic.2013.11.003
196	Mehta M, Pastor CA, Shah B. Achieving optimal influenza vaccination rates: a survey-based study of healthcare workers in an urban hospital. J Hosp Infect. 2008;70(1):76-79. doi:10.1016/j.jhin.2008.04.028
197	Khan TM, Khan AU, Ali I, Wu DB. Knowledge, attitude and awareness among healthcare professionals about influenza vaccination in Peshawar, Pakistan. Vaccine. 2016;34(11):1393-1398. doi:10.1016/j.vaccine.2016.01.045
198	Oria PA, Matini W, Nelligan I, et al. Are Kenyan healthcare workers willing to receive the pandemic influenza vaccine? Results from a cross-sectional survey of healthcare workers in Kenya about knowledge, attitudes and practices concerning infection with and vaccination against 2009 pandemic influenza A (H1N1), 2010. Vaccine. 2011;29(19):3617-3622. doi:10.1016/j.vaccine.2011.01.063
199	Benedict Kpozehouen E, Arrudsivah B, Tan TC, Raina Macintyre C. Knowledge, attitudes and practices of health care workers in a cardiology department on influenza vaccination. Vaccine. 2023;41(14):2349-2356. doi:10.1016/j.vaccine.2023.01.070
200	Jiang B, Cao Y, Qian J, et al. Healthcare Workers' Attitudes toward Influenza Vaccination: A Behaviour and Social Drivers Survey. Vaccines (Basel). 2023;11(1):143. Published 2023 Jan 9. doi:10.3390/vaccines11010143
201	Galanis P, Katsiroumpa A, Vraka I, et al. Seasonal Influenza Vaccine Intention among Nurses Who Have Been Fully Vaccinated against COVID-19: Evidence from Greece. Vaccines (Basel). 2023;11(1):159. Published 2023 Jan 11. doi:10.3390/vaccines11010159
202	Yang X, Tang W, Tan Q, Mao D, Ding X. The Vaccine Hesitancy Profiles and Determinants of Seasonal Influenza among Chinese Community Healthcare Workers: A Cross-Sectional Study. Vaccines (Basel). 2022;10(9):1547. Published 2022 Sep 16. doi:10.3390/vaccines10091547
203	Sallam M, Ghazy RM, Al-Salahat K, et al. The Role of Psychological Factors and Vaccine Conspiracy Beliefs in Influenza Vaccine Hesitancy and Uptake among Jordanian Healthcare Workers during the COVID-19 Pandemic. Vaccines (Basel). 2022;10(8):1355. Published 2022 Aug 19. doi:10.3390/vaccines10081355

204	Nowalk MP, Lin CJ, Zimmerman RK, et al. Establish the habit: influenza vaccination for health care personnel. J Healthc Qual. 2010;32(2):35-42. doi:10.1111/j.1945-1474.2010.00073.x
205	Walker DW, Sloan SS, Kozlica JD. Public health worker attitudes and beliefs concerning 2009 H1N1 and seasonal influenza vaccines. Am J Infect Control. 2012;40(3):267-269. doi:10.1016/j.ajic.2011.05.010
206	Nowalk MP, Lin CJ, Zimmerman RK, et al. Self-reported influenza vaccination rates among health care workers in a large health system. Am J Infect Control. 2008;36(8):574-581. doi:10.1016/j.ajic.2008.01.008
207	Ojha RP, Stallings-Smith S, Flynn PM, Adderson EE, Offutt-Powell TN, Gaur AH. The Impact of Vaccine Concerns on Racial/Ethnic Disparities in Influenza Vaccine Uptake Among Health Care Workers. Am J Public Health. 2015;105(9):e35-e41. doi:10.2105/AJPH.2015.302736
208	Lehmann BA, Ruiter RA, Wicker S, van Dam D, Kok G. "I don't see an added value for myself": a qualitative study exploring the social cognitive variables associated with influenza vaccination of Belgian, Dutch and German healthcare personnel. BMC Public Health. 2014;14:407. Published 2014 Apr 28. doi:10.1186/1471-2458-14-407
209	Ko K, Kim S, Kim SH, Son KY, Lee J, Lee DR. Knowledge, Current Status, and Barriers toward Healthcare Worker Vaccination among Family Medicine Resident Participants in a Web-Based Survey in Korea. Korean J Fam Med. 2017;38(1):21-27. doi:10.4082/kjfm.2017.38.1.21
210	Evren H, Evren EÜ, Özcem SB, Yazgan ZÖ, Barış SA, Yıldız F. The Knowledge Level of Hospital Staff about Influenza and Pneumococcal Vaccination. Cyprus J Med Sci. 2019;4(3):220-4.
211	Pullagura GR, Violette R, Houle SKD, Waite NM. Exploring influenza vaccine hesitancy in community pharmacies: Knowledge, attitudes and practices of community pharmacists in Ontario, Canada. Can Pharm J (Ott). 2020;153(6):361-370. Published 2020 Oct 5. doi:10.1177/1715163520960744
212	Smith S, Sim J, Halcomb E. Australian general practice nurse's knowledge, attitudes and practices regarding influenza vaccination: a cross-sectional survey. J Clin Nurs. 2016;25(17-18):2502-2510. doi:10.1111/jocn.13287
213	Sundaram N, Duckett K, Yung CF, et al. "I wouldn't really believe statistics" - Challenges with influenza vaccine acceptance among healthcare workers in Singapore. Vaccine. 2018;36(15):1996-2004. doi:10.1016/j.vaccine.2018.02.102
214	Haridi HK, Salman KA, Basaif EA, Al-Skaibi DK. Influenza vaccine uptake, determinants, motivators, and barriers of the vaccine receipt among healthcare workers in a tertiary care hospital in Saudi Arabia. J Hosp Infect. 2017;96(3):268-275. doi:10.1016/j.jhin.2017.02.005
215	Waheed A, Waheeb Y, Hassan A, Fahim A. Seasonal influenza vaccination coverage and barriers among healthcare workers in an Egyptian Province. Med Lav. 2020;111(6):449-456. Published 2020 Oct 15. doi:10.23749/mdl.v111i6.9489
216	Halaseh L, Yasein N. Influenza vaccine: immunization rates, knowledge and attitudes of healthcare workers in Jordan. Kuwait Med J. 2020 Jun 1;52:156-63.
217	Hussain H, McGeer A, McNeil S, et al. Factors associated with influenza vaccination among healthcare workers in acute care hospitals in Canada. Influenza Other Respir Viruses. 2018;12(3):319-325. doi:10.1111/irv.12545

218	Lau LHW, Lee SS, Wong NS. The continuum of influenza vaccine hesitancy among nursing professionals in Hong Kong. Vaccine. 2020;38(43):6785-6793. doi:10.1016/j.vaccine.2020.08.038
219	Neufeind J, Wenchel R, Boedeker B, Wicker S, Wichmann O. Monitoring influenza vaccination coverage and acceptance among health-care workers in German hospitals - results from three seasons. Hum Vaccin Immunother. 2021;17(3):664-672. doi:10.1080/21645515.2020.1801072
220	Alqahtani FM, Kamel SA, Almudarra S, Mathkour AA. Vaccination against Influenza among Health Care Workers in Al Mashaer during Hajj 2019 (1440 H); Uptake and Barriers. Middle East Journal of Family Medicine. 2022 Aug 1;7(10):112.
221	Ali I, Ijaz M, Rehman IU, Rahim A, Ata H. Knowledge, Attitude, Awareness, and Barriers Toward Influenza Vaccination Among Medical Doctors at Tertiary Care Health Settings in Peshawar, Pakistan-A Cross-Sectional Study. Front Public Health. 2018;6:173. Published 2018 Jun 27. doi:10.3389/fpubh.2018.00173
222	Bonaccorsi G, Lorini C, Santomauro F, et al. Predictive factors associated with the acceptance of pandemic and seasonal influenza vaccination in health care workers and students in Tuscany, Central Italy. Hum Vaccin Immunother. 2013;9(12):2603-2612. doi:10.4161/hv.26036
223	Kan T, Ai J, Zhang J, Liu X. Predictors of seasonal influenza vaccination behaviour among nurses and implications for interventions to increase vaccination uptake: A cross-sectional survey. Int J Nurs Stud. 2018;79:137-144. doi:10.1016/j.ijnurstu.2017.12.003
224	Honda H, Sato Y, Yamazaki A, Padival S, Kumagai A, Babcock H. A successful strategy for increasing the influenza vaccination rate of healthcare workers without a mandatory policy outside of the United States: a multifaceted intervention in a Japanese tertiary care center. Infect Control Hosp Epidemiol. 2013;34(11):1194-1200. doi:10.1086/673452
225	Vieira RH, Erdmann AL, Andrade SR, Freitas PF. Influenza vaccination among nursing professionals: reality and challenges. Acta Paulista de Enfermagem. 2012;25:104-9.
226	Pless A, McLennan SR, Nicca D, Shaw DM, Elger BS. Reasons why nurses decline influenza vaccination: a qualitative study. BMC Nurs. 2017;16:20. Published 2017 Apr 28. doi:10.1186/s12912-017-0215-5
227	Rong H, Lai X, Ma X, et al. Seasonal Influenza Vaccination and Recommendation: The Difference between General Practitioners and Public Health Workers in China. Vaccines (Basel). 2020;8(2):265. Published 2020 May 31. doi:10.3390/vaccines8020265
228	Lindvig SO, Larsen L. Uptake of and attitudes towards influenza vaccination among Danish hospital healthcare workers. Dan Med J. 2021;68(3):A10200729. Published 2021 Feb 16.
229	Khalooei A, Bafti MS. Factors Associated with Seasonal Influenza Vaccination Among Nursing Staff of Three Teaching Hospitals Affiliated to Kerman University of Medical Sciences, Iran. Shiraz E-Medical Journal. 2021 May 31;22(5).
230	Robbins T, Berry L, Wells F, Randeva H, Laird S. Healthcare staff perceptions towards influenza and potential COVID-19 vaccination in the 2020 pandemic context. J Hosp Infect. 2021;112:45-48. doi:10.1016/j.jhin.2021.02.024
231	Di Giuseppe G, Pelullo CP, Paolantonio A, Della Polla G, Pavia M. Healthcare Workers' Willingness to Receive Influenza Vaccination in the Context of the COVID-19 Pandemic: A Survey in Southern Italy. Vaccines (Basel). 2021;9(7):766. Published 2021 Jul 9. doi:10.3390/vaccines9070766

232	Quintyne KI, Daly E, Brabazon E, Finnegan P, Kavanagh P. Attitudes and Uptake of Seasonal Influenza Vaccination for Health Service Executive (HSE) Staff. Ir Med J. 2018;111(5):749. Published 2018 May 10.
233	Hussain NA, Al-Yasseri BJ. Practicing, Attitudes and Beliefs of Physicians at Al-Emamain Medical City towards Seasonal Influenza Vaccination. Indian Journal of Public Health. 2019 Oct;10(10):3127.
234	AlMarzooqi LM, AlMajidi AA, AlHammadi AA, AlAli N, Khansaheb HH. Knowledge, attitude, and practice of influenza vaccine immunization among primary healthcare providers in Dubai health authority, 2016-2017. Hum Vaccin Immunother. 2018;14(12):2999-3004. doi:10.1080/21645515.2018.1507667
235	Moser A, Mabire C, Hugli O, et al. Vaccination Against Seasonal or Pandemic Influenza in Emergency Medical Services. Prehosp Disaster Med. 2016;31(2):155-162. doi:10.1017/S1049023X16000121
236	Tuckerman JL, Shrestha L, Collins JE, Marshall HS. Understanding motivators and barriers of hospital-based obstetric and pediatric health care worker influenza vaccination programs in Australia. Hum Vaccin Immunother. 2016;12(7):1749-1756. doi:10.1080/21645515.2016.1153204
237	Costantino C, Amodio E, Calamusa G, Vitale F, Mazzucco W. Could university training and a proactive attitude of coworkers be associated with influenza vaccination compliance? A multicentre survey among Italian medical residents. BMC Med Educ. 2016;16:38. Published 2016 Jan 29. doi:10.1186/s12909-016-0558-8
238	Nutman A, Yoeli N. Influenza vaccination motivators among healthcare personnel in a large acute care hospital in Israel. Isr J Health Policy Res. 2016;5:52. Published 2016 Oct 26. doi:10.1186/s13584-016-0112-5
239	Hwisa NT, Katakam P, Chandu BR, Ismael MH, Bader A. Pandemic influenza A (H1N1) vaccination among libyan health care personnel: A cross-sectional retrospective study. J Pharm Bioallied Sci. 2014;6(3):192-197. doi:10.4103/0975-7406.130958
240	Marcu A, Rubinstein H, Michie S, Yardley L. Accounting for personal and professional choices for pandemic influenza vaccination amongst English healthcare workers. Vaccine. 2015;33(19):2267-2272. doi:10.1016/j.vaccine.2015.03.028
241	Ryser AJ, Heininger U. Comparative acceptance of pertussis and influenza immunization among health-care personnel. Vaccine. 2015;33(41):5350-5356. doi:10.1016/j.vaccine.2015.08.078
242	Tuckerman JL, Collins JE, Marshall HS. Factors affecting uptake of recommended immunizations among health care workers in South Australia. Hum Vaccin Immunother. 2015;11(3):704-712. doi:10.1080/21645515.2015.1008886
243	Satman I, Akalin S, Cakir B, Altinel S; diaVAX Study Group. The effect of physicians' awareness on influenza and pneumococcal vaccination rates and correlates of vaccination in patients with diabetes in Turkey: an epidemiological Study "diaVAX". Hum Vaccin Immunother. 2013;9(12):2618-2626. doi:10.4161/hv.25826
244	Antón-Ladislao A, García-Gutiérrez S, Soldevila N, et al. Visualizing knowledge and attitude factors related to influenza vaccination of physicians. Vaccine. 2015;33(7):885-891. doi:10.1016/j.vaccine.2014.12.012
245	Zhang J, While AE, Norman IJ. Nurses' knowledge and risk perception towards seasonal influenza and vaccination and their vaccination behaviours: a cross-sectional survey. Int J Nurs Stud. 2011;48(10):1281-1289. doi:10.1016/j.ijnurstu.2011.03.002
246	Aziz NA, Muhamad S, Manaf MR, Hamid MZ. Factors Influencing H1N1 Vaccination Among Primary Health Care Workers: A Cross-Sectional Study. Int J Prev Med. 2013;4(6):664-670.

247	Mytton OT, O'Moore EM, Sparkes T, Baxi R, Abid M. Knowledge, attitudes and beliefs of health care workers towards influenza vaccination. Occup Med (Lond). 2013;63(3):189-195. doi:10.1093/occmed/kqt002
248	Chen SC, Hawkins G, Aspinall E, Patel N. Factors influencing uptake of influenza A (H1N1) vaccine amongst healthcare workers in a regional pediatric centre: lessons for improving vaccination rates. Vaccine. 2012;30(2):493-497. doi:10.1016/j.vaccine.2011.04.032
249	Schult TM, Awosika ER, Hodgson MJ, et al. Innovative approaches for understanding seasonal influenza vaccine declination in healthcare personnel support development of new campaign strategies. Infect Control Hosp Epidemiol. 2012;33(9):924-931. doi:10.1086/667370
250	Parry HM, Damery S, Fergusson A, Draper H, Bion J, Low AE. Pandemic influenza A (H1N1) 2009 in a critical care and theatre setting: beliefs and attitudes towards staff vaccination. J Hosp Infect. 2011;78(4):302-307. doi:10.1016/j.jhin.2011.02.009
251	Supranowicz P, Brydak LB. Opinions of employees of the National Institute of Public Health--National Institute of Hygiene in Warsaw on influenza vaccination. Przegl Epidemiol. 2013;67(4):667-760.
252	Wicker S, Rabenau HF, Betz W, Lauer HC. Attitudes of dental healthcare workers towards the influenza vaccination. Int J Hyg Environ Health. 2012;215(4):482-486. doi:10.1016/j.ijheh.2011.08.005
253	Thompson MG, Gaglani MJ, Naleway A, et al. The expected emotional benefits of influenza vaccination strongly affect pre-season intentions and subsequent vaccination among healthcare personnel. Vaccine. 2012;30(24):3557-3565. doi:10.1016/j.vaccine.2012.03.062
254	Mistik S, Balci E, Elmali F. Primary healthcare professionals' knowledge, attitude and behavior regarding influenza immunization; 2006-2007 season adverse effect profile. Bratisl Lek Listy. 2012;113(6):384-388. doi:10.4149/bll_2012_088
255	Sevencan F, Ertem M, Özçullu N, Dorman V, Kubat NK. The evaluation of the opinions and attitudes of healthcare personnel of the province Diyarbakir against influenza A (H1N1) and the vaccination. Hum Vaccin. 2011;7(9):945-951. doi:10.4161/hv.7.9.16368
256	López-Picado A, Apiñaniz A, Ramos AL, et al. Knowledge, attitudes and perceptions of health professionals in relation to A/H1N1 influenza and its vaccine. Emerg Health Threats J. 2012;5:10.3402/ehtj.v5i0.7266. doi:10.3402/ehtj.v5i0.7266
257	Hu SS, Yang LL, Chen SH, Wang XF, Han YF, Zhang WF. Intention to accept pandemic H1N1 vaccine and the actual vaccination coverage in nurses at a Chinese children’s hospital. Hong Kong J Paediatr. 2011 Apr 1;16:101-6.
258	Wicker S, Rabenau HF. A patient safety issue: Mandatory influenza vaccination for health care workers. Procedia in Vaccinology. 2010 Jan 1;2(1):101-5.
259	Dedoukou X, Nikolopoulos G, Maragos A, Giannoulidou S, Maltezou HC. Attitudes towards vaccination against seasonal influenza of health-care workers in primary health-care settings in Greece. Vaccine. 2010;28(37):5931-5933. doi:10.1016/j.vaccine.2010.06.108
260	Thoon KC, Chong CY. Survey of healthcare workers' attitudes, beliefs and willingness to receive the 2009 pandemic influenza A (H1N1) vaccine and the impact of educational campaigns. Ann Acad Med Singap. 2010;39(4):.
261	Douville LE, Myers A, Jackson MA, Lantos JD. Health care worker knowledge, attitudes, and beliefs regarding mandatory influenza vaccination. Arch Pediatr Adolesc Med. 2010;164(1):33-37. doi:10.1001/archpediatrics.2009.252

262	Polat HH, Yalçin AN, Öncel S. Influenza vaccination; Rates, knowledge and the attitudes of physicians in a university hospital. Turkiye Klin J Med Sci. 2010;30:48-53.
263	Bautista D, Vila B, Uso R, Tellez M, Zanon V. Predisposing, reinforcing, and enabling factors influencing influenza vaccination acceptance among healthcare workers. Infect Control Hosp Epidemiol. 2006;27(1):73-77. doi:10.1086/499148
264	Bishburg E, Shah M, Mathis AS. Influenza vaccination among medical residents in a teaching hospital . Infect Control Hosp Epidemiol. 2008;29(1):89-91. doi:10.1086/524322
265	Livni G, Chodik G, Yaari A, Tirosh N, Ashkenazi S. Attitudes, knowledge and factors related to acceptance of influenza vaccine by pediatric healthcare workers. Journal of pediatric infectious diseases. 2008 Jan 1;3(2):111-7.
266	Maltezou HC, Maragos A, Katerelos P, et al. Influenza vaccination acceptance among health-care workers: a nationwide survey. Vaccine. 2008;26(11):1408-1410. doi:10.1016/j.vaccine.2008.01.049
267	Lorick SA, Wortley PM, Lindley MC, Bardenheier BH, Euler GL. U.S. Healthcare personnel and influenza vaccination during the 2004-2005 vaccine shortage. Am J Prev Med. 2008;34(6):455-462. doi:10.1016/j.amepre.2008.01.031
268	Esposito S, Tremolati E, Bellasio M, et al. Attitudes and knowledge regarding influenza vaccination among hospital health workers caring for women and children. Vaccine. 2007;25(29):5283-5289. doi:10.1016/j.vaccine.2007.05.011
269	Nafziger DA, Herwaldt LA. Attitudes of internal medicine residents regarding influenza vaccination. Infect Control Hosp Epidemiol. 1994;15(1):32-35. doi:10.1086/646815
270	Campos W, Jalaludin BB. Predictors of influenza vaccination amongst Australian nurses. Aust J Adv Nurs. 2002;20(2):19-21.
271	Serwint J. Pediatrician-dependent barriers in influenza vaccine administration. Pediatr Infect Dis J. 1993;12(11):956-958. doi:10.1097/00006454-199311000-00014
272	Wodi AP, Samy S, Ezeanolue E, et al. Influenza vaccine: immunization rates, knowledge, and attitudes of resident physicians in an urban teaching hospital. Infect Control Hosp Epidemiol. 2005;26(11):867-873. doi:10.1086/502510
273	Kaya A, Altınel N, Karakaya G, Çetinkaya F. Knowledge and attitudes among patients with asthma and parents and physicians towards influenza vaccination. Allergol Immunopathol (Madr). 2017;45(3):240-243. doi:10.1016/j.aller.2016.08.003
274	Alhalaseh L, Fayoumi H, Khalil B. The Health Belief Model in predicting healthcare workers' intention for influenza vaccine uptake in Jordan. Vaccine. 2020;38(46):7372-7378. doi:10.1016/j.vaccine.2020.09.002
275	Hothersall EJ, de Bellis-Ayres S, Jordan R. Factors associated with uptake of pandemic influenza vaccine among general practitioners and practice nurses in Shropshire, UK. Prim Care Respir J. 2012;21(3):302-307. doi:10.4104/pcrj.2012.00056
276	Verger P, Flicoteaux R, Schwarzinger M, et al. Pandemic influenza (A/H1N1) vaccine uptake among French private general practitioners: a cross sectional study in 2010. PLoS One. 2012;7(8):e41837. doi:10.1371/journal.pone.0041837
277	Al-Tawfiq JA, Antony A, Abed MS. Attitudes towards influenza vaccination of multi-nationality health-care workers in Saudi Arabia. Vaccine. 2009;27(40):5538-5541. doi:10.1016/j.vaccine.2009.06.108
278	Hopman CE, Riphagen-Dalhuisen J, Looijmans-van den Akker I, et al. Determination of factors required to increase uptake of influenza vaccination among hospital-based healthcare workers. J Hosp Infect. 2011;77(4):327-331. doi:10.1016/j.jhin.2010.10.009

279	Alame M, Kaddoura M, Kharroubi S, et al. Uptake rates, knowledge, attitudes, and practices toward seasonal influenza vaccination among healthcare workers in Lebanon. Hum Vaccin Immunother. 2021;17(11):4623-4631. doi:10.1080/21645515.2021.1948783
280	Talbot TR, Dellit TH, Hebden J, Sama D, Cuny J. Factors associated with increased healthcare worker influenza vaccination rates: results from a national survey of university hospitals and medical centers. Infect Control Hosp Epidemiol. 2010;31(5):456-462. doi:10.1086/651666
281	Samyn M, Van Hal G, Vandevijvere H, Van Damme P. The influenza vaccine for nursing and care professionals at emergency services in Flanders. Hum Vaccin Immunother. 2021;17(8):2719-2722. doi:10.1080/21645515.2021.1894062
282	Arghittu A, Dettori M, Azara A, et al. Flu Vaccination Attitudes, Behaviours, and Knowledge among Health Workers. Int J Environ Res Public Health. 2020;17(9):3185. Published 2020 May 3. doi:10.3390/ijerph17093185
283	Durovic A, Widmer AF, Dangel M, Ulrich A, Battegay M, Tschudin-Sutter S. Low rates of influenza vaccination uptake among healthcare workers: Distinguishing barriers between occupational groups. Am J Infect Control. 2020;48(10):1139-1143. doi:10.1016/j.ajic.2020.02.004
284	Alabbad AA, Alsaad AK, Al Shaalan MA, Alola S, Albanyan EA. Prevalence of influenza vaccine hesitancy at a tertiary care hospital in Riyadh, Saudi Arabia. J Infect Public Health. 2018;11(4):491-499. doi:10.1016/j.jiph.2017.09.002
285	Rabaan AA, Wyse R, Al-Tawfiq JA, et al. Influenza vaccine acceptance by healthcare workers in Saudi Arabia: A questionnaire-based analysis. Infez Med. 2020;28(1):70-77.
286	Ward K, Seale H, Zwar N, Leask J, Macintyre CR. Annual influenza vaccination: coverage and attitudes of primary care staff in Australia. Influenza Other Respir Viruses. 2011;5(2):135-141. doi:10.1111/j.1750-2659.2010.00158.x
287	Clark SJ, Cowan AE, Wortley PM. Influenza vaccination attitudes and practices among US registered nurses. Am J Infect Control. 2009;37(7):551-556. doi:10.1016/j.ajic.2009.02.012
288	Albano L, Matuozzo A, Marinelli P, Di Giuseppe G. Knowledge, attitudes and behaviour of hospital health-care workers regarding influenza A/H1N1: a cross sectional survey. BMC Infect Dis. 2014;14:208. Published 2014 Apr 16. doi:10.1186/1471-2334-14-208
289	Sočan M, Erčulj V, Lajovic J. Knowledge and attitudes on pandemic and seasonal influenza vaccination among Slovenian physicians and dentists. Eur J Public Health. 2013;23(1):92-97. doi:10.1093/eurpub/cks006
290	Smedley J, Poole J, Waclawski E, et al. Influenza immunisation: attitudes and beliefs of UK healthcare workers. Occup Environ Med. 2007;64(4):223-227. doi:10.1136/oem.2005.023564
291	Gramegna A, Dellafiore S, Contarini M, et al. Knowledge and attitudes on influenza vaccination among Italian physicians specialized in respiratory infections: an Italian Respiratory Society (SIP/IRS) web-based survey [published correction appears in J Prev Med Hyg. 2018 Sep 28;59(3):E248]. J Prev Med Hyg. 2018;59(2):E128-E131. Published 2018 Jun 1.
292	Riccò M, Cattani S, Casagranda F, Gualerzi G, Signorelli C. Knowledge, attitudes, beliefs and practices of Occupational Physicians towards seasonal influenza vaccination: a cross-sectional study from North-Eastern Italy. J Prev Med Hyg. 2017;58(2):E141-E154.
293	Alhammadi A, Khalifa M, Abdulrahman H, Almuslemani E, Alhothi A, Janahi M. Attitudes and perceptions among the pediatric health care providers toward influenza vaccination in Qatar: A cross-sectional study. Vaccine. 2015;33(32):3821-3828. doi:10.1016/j.vaccine.2015.06.082

294	Hosamirudsari H, Kanavee AR, Ghanbari M, Akbarpour S, Alimohamadi Y. Assessment of the belief and attitudes of Iranian healthcare personnel's toward the influenza infection and influenza vaccination. J Prev Med Hyg. 2019;60(3):E178-E183. Published 2019 Sep 30. doi:10.15167/2421-4248/jpmh2019.60.3.1056
295	James PB, Rehman IU, Bah AJ, Lahai M, Cole CP, Khan TM. An assessment of healthcare professionals' knowledge about and attitude towards influenza vaccination in Freetown Sierra Leone: a cross-sectional study. BMC Public Health. 2017;17(1):692. Published 2017 Sep 5. doi:10.1186/s12889-017-4700-2
296	Alkuwari MG, Aziz NA, Nazzal ZA, Al-Nuaimi SA. Pandemic influenza A/H1N1 vaccination uptake among health care workers in Qatar: motivators and barriers. Vaccine. 2011;29(11):2206-2211. doi:10.1016/j.vaccine.2010.08.093
297	Jung Y, Kwon M, Song J. Stepwise intervention including 1-on-1 counseling is highly effective in increasing influenza vaccination among health care workers. Am J Infect Control. 2017;45(6):635-641. doi:10.1016/j.ajic.2016.11.012
298	Thomas DR, Winsted B, Koontz C. Improving neglected influenza vaccination among healthcare workers in long-term care. J Am Geriatr Soc. 1993;41(9):928-930. doi:10.1111/j.1532-5415.1993.tb06757.x
299	LaVela SL, Smith B, Weaver FM, Legro MW, Goldstein B, Nichol K. Attitudes and practices regarding influenza vaccination among healthcare workers providing services to individuals with spinal cord injuries and disorders. Infect Control Hosp Epidemiol. 2004;25(11):933-940. doi:10.1086/502323
300	Vírseda S, Restrepo MA, Arranz E, et al. Seasonal and Pandemic A (H1N1) 2009 influenza vaccination coverage and attitudes among health-care workers in a Spanish University Hospital. Vaccine. 2010;28(30):4751-4757. doi:10.1016/j.vaccine.2010.04.101
301	Bryant KA, Stover B, Cain L, Levine GL, Siegel J, Jarvis WR. Improving influenza immunization rates among healthcare workers caring for high-risk pediatric patients. Infect Control Hosp Epidemiol. 2004;25(11):912-917. doi:10.1086/502319
302	Abramson ZH, Levi O. Influenza vaccination among primary healthcare workers. Vaccine. 2008;26(20):2482-2489. doi:10.1016/j.vaccine.2008.03.011
303	Kenny E, McNamara Á, Noone C, Byrne M. Barriers to seasonal influenza vaccine uptake among health care workers in long-term care facilities: A cross-sectional analysis. Br J Health Psychol. 2020;25(3):519-539. doi:10.1111/bjhp.12419
304	Cozza V, Alfonsi V, Rota MC, Paolini V, Ciofi degli Atti ML. Promotion of influenza vaccination among health care workers: findings from a tertiary care children's hospital in Italy. BMC Public Health. 2015;15:697. Published 2015 Jul 24. doi:10.1186/s12889-015-2067-9
305	Sibanda M, Meyer JC, Godman B, Burnett RJ. Low influenza vaccine uptake by healthcare workers caring for the elderly in South African old age homes and primary healthcare facilities. BMC Public Health. 2023;23(1):91. Published 2023 Jan 12. doi:10.1186/s12889-022-14926-8
306	Head S, Atkin S, Allan K, Ferguson C, Lutchmun S, Cordery R. Vaccinating health care workers during an influenza pandemic. Occup Med (Lond). 2012;62(8):651-654. doi:10.1093/occmed/kqs098
307	Watanakunakorn C, Ellis G, Gemmel D. Attitude of healthcare personnel regarding influenza immunization. Infect Control Hosp Epidemiol. 1993;14(1):17-20.

308	Wong SY, Wong EL, Chor J, et al. Willingness to accept H1N1 pandemic influenza vaccine: a cross-sectional study of Hong Kong community nurses. BMC Infect Dis. 2010;10:316. Published 2010 Oct 29. doi:10.1186/1471-2334-10-316
309	Qureshi AM, Hughes NJ, Murphy E, Primrose WR. Factors influencing uptake of influenza vaccination among hospital-based health care workers. Occup Med (Lond). 2004;54(3):197-201. doi:10.1093/occmed/kqg087
310	Conte A, Quattrin R, Filiputti E, et al. Promotion of flu vaccination among healthcare workers in an Italian academic hospital: An experience with tailored web tools. Hum Vaccin Immunother. 2016;12(10):2628-2633. doi:10.1080/21645515.2016.1186319
311	Palmore TN, Vandersluis JP, Morris J, et al. A successful mandatory influenza vaccination campaign using an innovative electronic tracking system. Infect Control Hosp Epidemiol. 2009;30(12):1137-1142. doi:10.1086/648084
312	Habib S, Rishpon S, Rubin L. Influenza vaccination among healthcare workers [published correction appears in Isr Med Assoc J. 2001 Jan;3(1):76]. Isr Med Assoc J. 2000;2(12):899-901.
313	Ballestas T, McEvoy SP, Doyle J; SMAHS Healthcare Worker Influenza Vaccination Working Party. Co-ordinated approach to healthcare worker influenza vaccination in an area health service. J Hosp Infect. 2009;73(3):203-209. doi:10.1016/j.jhin.2009.07.028
314	Ma L, Han X, Ma Y, et al. Decreased influenza vaccination coverage among Chinese healthcare workers during the COVID-19 pandemic. Infect Dis Poverty. 2022;11(1):105. Published 2022 Oct 8. doi:10.1186/s40249-022-01029-0
315	Quan K, Tehrani DM, Dickey L, et al. Voluntary to mandatory: evolution of strategies and attitudes toward influenza vaccination of healthcare personnel. Infect Control Hosp Epidemiol. 2012;33(1):63-70. doi:10.1086/663210
316	Abu-Gharbieh E, Fahmy S, Rasool BA, Khan S. Influenza vaccination: healthcare workers attitude in three Middle East countries. Int J Med Sci. 2010;7(5):319-325. Published 2010 Sep 21. doi:10.7150/ijms.7.319
317	Ajenjo MC, Woeltje KF, Babcock HM, Gemeinhart N, Jones M, Fraser VJ. Influenza vaccination among healthcare workers: ten-year experience of a large healthcare organization. Infect Control Hosp Epidemiol. 2010;31(3):233-240. doi:10.1086/650449
318	Petek D, Kamnik-Jug K. Motivators and barriers to vaccination of health professionals against seasonal influenza in primary healthcare. BMC Health Serv Res. 2018;18(1):853. Published 2018 Nov 14. doi:10.1186/s12913-018-3659-8
319	Umbreen G, Rehman A, Avais M, et al. Knowledge, Attitude, Practice and Barriers Associated with Influenza Vaccination among Health Care Professionals Working at Tertiary Care Hospitals in Lahore, Pakistan: A Multicenter Analytical Cross-Sectional Study. Vaccines (Basel). 2023;11(1):136. Published 2023 Jan 6. doi:10.3390/vaccines11010136
320	Riccò M, Vezzosi L, Marchesi F. Vaccinating Front-Line Healthcare Workers: Results of a Pre-Pandemic Cross-Sectional Study from North-Eastern Italy on First Responders. Vaccines (Basel). 2022;10(9):1492. Published 2022 Sep 7. doi:10.3390/vaccines10091492
321	Sani T, Morelli I, Sarti D, et al. Attitudes of Healthcare Workers toward Influenza Vaccination in the COVID-19 Era. Vaccines (Basel). 2022;10(6):883. Published 2022 May 31. doi:10.3390/vaccines10060883
322	Wong EL, Wong SY, Lee N, Cheung A, Griffiths S. Healthcare workers' duty concerns of working in the isolation ward during the novel H1N1 pandemic. J Clin Nurs. 2012;21(9-10):1466-1475. doi:10.1111/j.1365-2702.2011.03783.x
323	Kearns EC, Callanan I, O'Reilly A, et al. Changing attitudes towards annual influenza vaccination amongst staff in a Tertiary Care Irish University Hospital. Ir J Med Sci. 2022;191(2):629-636. doi:10.1007/s11845-021-02636-w

324	Barqawi H, Saddik B, Adra S, et al. Evaluating the knowledge, attitudes, and uptake of the influenza vaccine in healthcare professionals: A cross-sectional study from the United Arab Emirates. Pharm Pract (Granada). 2021;19(4):2587. doi:10.18549/PharmPract.2021.4.2587
325	Khazaeipour Z, Ranjbarnovin N, Hoseini N. Influenza immunization rates, knowledge, attitudes and practices of health care workers in Iran. J Infect Dev Ctries. 2010;4(10):636-644. Published 2010 Oct 28. doi:10.3855/jidc.1152
326	Weingarten S, Riedinger M, Bolton LB, Miles P, Ault M. Barriers to influenza vaccine acceptance. A survey of physicians and nurses. Am J Infect Control. 1989;17(4):202-207. doi:10.1016/0196-6553(89)90129-6
327	Smedley J, Palmer C, Baird J, Barker M. A survey of the delivery and uptake of influenza vaccine among health care workers. Occup Med (Lond). 2002;52(5):271-276. doi:10.1093/occmed/52.5.271
328	Awaidy STA, K Al Mayahi Z, Kaddoura M, et al. Influenza Vaccination Hesitancy among Healthcare Workers in South Al Batinah Governorate in Oman: A Cross-Sectional Study. Vaccines (Basel). 2020;8(4):661. Published 2020 Nov 6. doi:10.3390/vaccines8040661
329	Colaprico C, Ricci E, Bongiovanni A, et al. Flu Vaccination among Healthcare Professionals in Times of COVID-19: Knowledge, Attitudes, and Behavior. Vaccines (Basel). 2022;10(8):1341. Published 2022 Aug 18. doi:10.3390/vaccines10081341
330	Knowler P, Barrett M, Watson DA. Attitudes of healthcare workers to influenza vaccination. Infection, Disease & Health. 2018 Sep 1;23(3):156-62.
331	Palizi F, Mehrdad R, Izadi N. Influenza Vaccination attitudes of healthcare workers in Iran. Int J Pharm Technol. 2016;8:13978-86.
332	Mustafa M, Al-Khal A, Al Maslamani M, Al Soub H. Improving influenza vaccination rates of healthcare workers: a multipronged approach in Qatar. East Mediterr Health J. 2017;23(4):303-310. Published 2017 Jun 14. doi:10.26719/2017.23.4.303
333	Hulo S, Nuvoli A, Sobaszek A, Salembier-Trichard A. Knowledge and attitudes towards influenza vaccination of health care workers in emergency services. Vaccine. 2017;35(2):205-207. doi:10.1016/j.vaccine.2016.11.086
334	Domínguez A, Godoy P, Castilla J, et al. Knowledge of and attitudes to influenza in unvaccinated primary care physicians and nurses. Hum Vaccin Immunother. 2014;10(8):2378-2386. doi:10.4161/hv.29142
335	O'Neil CA, Kim L, Prill MM, et al. Preventing Respiratory Viral Transmission in Long-Term Care: Knowledge, Attitudes, and Practices of Healthcare Personnel. Infect Control Hosp Epidemiol. 2017;38(12):1449-1456. doi:10.1017/ice.2017.232
336	Christian MA. Influenza and hepatitis B vaccine acceptance: a survey of health care workers. Am J Infect Control. 1991;19(4):177-184. doi:10.1016/0196-6553(91)90001-s
337	Costantino C, Mazzucco W, Azzolini E, et al. Influenza vaccination coverage among medical residents: an Italian multicenter survey. Hum Vaccin Immunother. 2014;10(5):1204-1210. doi:10.4161/hv.28081
338	Thompson MG, McIntyre AF, Naleway AL, et al. Potential influence of seasonal influenza vaccination requirement versus traditional vaccine promotion strategies on unvaccinated healthcare personnel. Vaccine. 2013;31(37):3915-3921. doi:10.1016/j.vaccine.2013.06.045
339	Edwards F, Masick KD, Armellino D. Impact of the flu mask regulation on health care personnel influenza vaccine acceptance rates. Am J Infect Control. 2016;44(10):1154-1157. doi:10.1016/j.ajic.2016.03.006

340	Lucerón CO, Sánchez AL, de la Fuente Sánchez M, Moreno EG, de Gripe GD. Influenza vaccine hesitancy among healthcare workers in Spain: Results of a survey. Vacunas (English Edition). 2022 Sep 1;23(3):174-82.
341	Jennings AR, Burant CJ. Influenza vaccination knowledge and perceptions among Veterans Affairs nurses. Am J Infect Control. 2013;41(8):737-739. doi:10.1016/j.ajic.2013.01.005
342	Rachiotis G, Mouchtouri VA, Kremastinou J, Gourgoulianis K, Hadjichristodoulou C. Low acceptance of vaccination against the 2009 pandemic influenza A(H1N1) among healthcare workers in Greece. Euro Surveill. 2010;15(6):19486. Published 2010 Feb 11.
343	Askarian M, Khazaeipour Z, McLaws ML. Facilitators for influenza vaccination uptake in nurses at the Shiraz University of Medical Sciences. Public Health. 2011;125(8):512-517. doi:10.1016/j.puhe.2011.03.012
344	Dubé E, Gagnon D, Kiely M, Boulianne N, Landry M. Acceptability of live attenuated influenza vaccine by vaccine providers in Quebec, Canada. Hum Vaccin Immunother. 2015;11(4):956-960. doi:10.1080/21645515.2015.1009816
345	Elbarazi I, Al-Hamad S, Alfalasi S, Aldhaheri R, Dubé E, Alsuwaidi AR. Exploring vaccine hesitancy among healthcare providers in the United Arab Emirates: a qualitative study. Hum Vaccin Immunother. 2021;17(7):2018-2025. doi:10.1080/21645515.2020.1855953
346	Edwards F, Armellino D. What is the Impact of the 'Flu Mask'Regulation on Healthcare Personnel Influenza Vaccine Acceptance Rates?. American Journal of Infection Control. 2015 Jun 2;43(6):S43.
347	Alshammari TM, Yusuff KB, Aziz MM, Subaie GM. Healthcare professionals' knowledge, attitude and acceptance of influenza vaccination in Saudi Arabia: a multicenter cross-sectional study. BMC Health Serv Res. 2019;19(1):229. Published 2019 Apr 15. doi:10.1186/s12913-019-4054-9
348	Moore BS. Why health care workers decline influenza vaccination. AAOHN J. 2009;57(11):475-478. doi:10.3928/08910162-20091027-02
349	Osman AD. Reasons for and barriers to influenza vaccination among healthcare workers in an Australian emergency department. Australian Journal of Advanced Nursing, The. 2010 Mar;27(3):38-43.
350	Nace DA, Hoffman EL, Resnick NM, Handler SM. Achieving and sustaining high rates of influenza immunization among long-term care staff. J Am Med Dir Assoc. 2007;8(2):128-133. doi:10.1016/j.jamda.2006.09.014
351	Neufeind J, Betsch C, Habersaat KB, Eckardt M, Schmid P, Wichmann O. Barriers and drivers to adult vaccination among family physicians - Insights for tailoring the immunization program in Germany. Vaccine. 2020;38(27):4252-4262. doi:10.1016/j.vaccine.2020.04.052
352	Johansen LJ, Stenvig T, Wey H. The decision to receive influenza vaccination among nurses in North and South Dakota. Public Health Nurs. 2012;29(2):116-125. doi:10.1111/j.1525-1446.2011.00966.x
353	Abu Hammour W, Al-Saleh S. Knowledge, attitudes, and practice of healthcare workers toward influenza vaccination at Al Jalila Children's Specialty Hospital (AJCH), Dubai, UAE. Int J Pediatr Adolesc Med. 2019;6(1):16-20. doi:10.1016/j.ijpam.2019.01.001
354	Kadi Z, Atif ML, Brenet A, Izoard S, Astagneau P. Barriers of influenza vaccination in health care personnel in France. Am J Infect Control. 2016;44(3):361-362. doi:10.1016/j.ajic.2015.09.027

355	Kitt E, Burt S, Price SM, et al. Implementation of a Mandatory Influenza Vaccine Policy: A 10-Year Experience. Clin Infect Dis. 2021;73(2):e290-e296. doi:10.1093/cid/ciaa782
356	Opstelten W, van Essen GA, Heijnen ML, Ballieux MJ, Goudswaard AN. High vaccination rates for seasonal and pandemic (A/H1N1) influenza among healthcare workers in Dutch general practice. Vaccine. 2010;28(38):6164-6168. doi:10.1016/j.vaccine.2010.07.031
357	Riphagen-Dalhuisen J, Frijstein G, van der Geest-Blankert N, et al. Planning and process evaluation of a multi-faceted influenza vaccination implementation strategy for health care workers in acute health care settings. BMC Infect Dis. 2013;13:235. Published 2013 May 23. doi:10.1186/1471-2334-13-235
358	Chittaro M, Turello D, Calligaris L, et al. Impact of vaccinating HCWs on the ward and possible influence of avian flu threat. Infection. 2009;37(1):29-33. doi:10.1007/s15010-008-8002-6
359	Gilardi F, Castelli Gattinara G, Vinci MR, et al. Seasonal Influenza Vaccination in Health Care Workers. A Pre-Post Intervention Study in an Italian Paediatric Hospital. Int J Environ Res Public Health. 2018;15(5):841. Published 2018 Apr 24. doi:10.3390/ijerph15050841
360	Dubé E, Fannie D, Vladimir G, et al. A(H1N1) pandemic influenza and its prevention by vaccination: paediatricians' opinions before and after the beginning of the vaccination campaign. BMC Public Health. 2011;11:128. Published 2011 Feb 22. doi:10.1186/1471-2458-11-128
361	Rosenblum AJ, Wend CM, Huang R, Spangler S, Barnett DJ, Levy MJ. Factors Associated With Emergency Medical Services Providers' Acceptance of the Seasonal Influenza Vaccine. Disaster Med Public Health Prep. 2022;16(3):1099-1104. doi:10.1017/dmp.2021.44
362	Bertoni L, Roncadori A, Gentili N, et al. How has COVID-19 pandemic changed flu vaccination attitudes among an Italian cancer center healthcare workers?. Hum Vaccin Immunother. 2022;18(1):1978795. doi:10.1080/21645515.2021.1978795
363	Alkathlan M, Khalil R, Alhemaidani MF, et al. Trends, Uptake, and Predictors of Influenza Vaccination Among Healthcare Practitioners During the COVID-19 Pandemic Flu Season (2020) and the Following Season (2021) in Saudi Arabia. J Multidiscip Healthc. 2021;14:2527-2536. Published 2021 Sep 15. doi:10.2147/JMDH.S330029
364	Looijmans-van den Akker I, Marsaoui B, Hak E, van Delden JJ. Beliefs on mandatory influenza vaccination of health care workers in nursing homes: a questionnaire study from the Netherlands. J Am Geriatr Soc. 2009;57(12):2253-2256. doi:10.1111/j.1532-5415.2009.02560.x
365	Maurer J, Harris KM, Black CL, Euler GL. Support for seasonal influenza vaccination requirements among US healthcare personnel. Infect Control Hosp Epidemiol. 2012;33(3):213-221. doi:10.1086/664056
366	Baron-Epel O, Madjar B, Grefat R, Rishpon S. Trust and the demand for autonomy may explain the low rates of immunizations among nurses. Hum Vaccin Immunother. 2013;9(1):100-107. doi:10.4161/hv.22503
367	Hamilton RA, Krockow EM, Vekria P. Attitudes towards influenza and uptake of the flu vaccine: A survey of pharmacy staff working in English hospitals. Vaccine. 2021;39(19):2636-2642. doi:10.1016/j.vaccine.2021.03.091

368	Polgreen PM, Septimus E, Talbot TR, Beekmann SE, Helms C. Results of a national survey of infectious diseases specialists regarding influenza vaccination programs for healthcare workers. Infect Control Hosp Epidemiol. 2010;31(10):1063-1065. doi:10.1086/656382
